# Rapid and Accurate Estimation of Quantum Average Electron Densities Using Newly-Defined Atom Types

**DOI:** 10.1021/acsomega.5c03444

**Published:** 2025-08-15

**Authors:** Alya A. Arabi

**Affiliations:** † College of Medicine and Health Sciences, Department of Biochemistry and Molecular Biology, United Arab Emirates University, Al Ain, P.O. Box: 15551, United Arab Emirates

## Abstract

A new computational tool, the Average Electron Density Estimator (AED-Est), has been developed along with a new scheme for assigning atom type, the AAA scheme. This combination of the AED-Est and the AAA scheme is designed to rapidly estimate properties, including electron populations, volumes, and average electron density (AED) values, with high precision and an accuracy comparable to values computed at the quantum levels. AED-Est gave comparable results when using three different sets/subsets of various neutral molecules. The AED-Est reference values were obtained using 553 molecules, and then tested on a separate set of 101 molecules. The *R* ^2^ between the predicted values (obtained via the AED-Est tool) and the actual values (obtained via quantum simulations) reaches 0.99, and the RMSE values are at least 1 order of magnitude smaller than the average values. The new AAA scheme of defining atom types, which can be useful in designing new force fields, can significantly imporve the results compared to the general Amber force field, GAFF2, scheme. The AED-Est tool provided even better predictions of AED values for groups of atoms within a molecule, such as bioisosteric moieties, than for individual atoms. This has significant implications for fields such as drug discovery and the development of more effective therapeutics.

## Graphical abstract

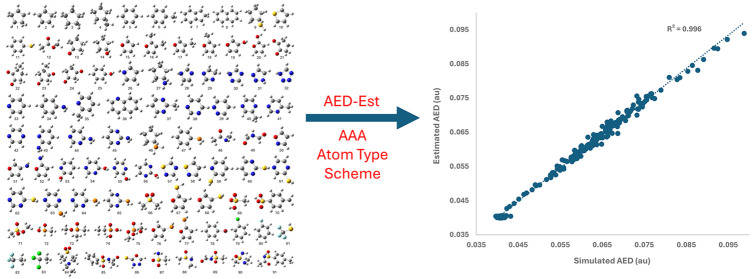

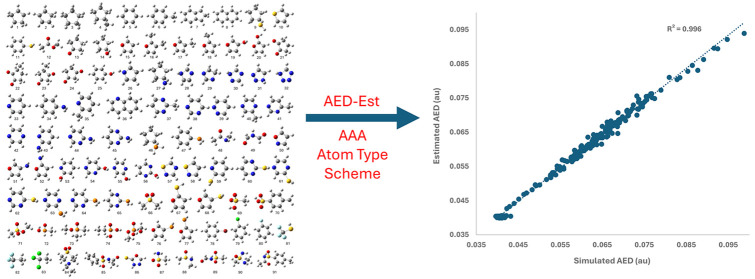

## Introduction

Recent advancements in quantum chemistry offer promising improvements in predictions related to drug design. In 2010, a quantum tool known as the Average Electron Density (AED) tool was developed.^1^ This tool, among others such as the Quantum Isostere Database (QID) tool,^2^ marked an advancement in the field of computer-aided drug design, particularly in bioisosterism. Bioisosteres are a part of a molecule that can be interchangeably used to modify the properties of pharmacologically active molecules while retaining their biological activity. This can help improve the efficacy, selectivity, or pharmacokinetic properties of a drug.^3^ Prior to the development of the AED tool, which enables the quantitative evaluation of bioisosterism, qualitative electrostatic potential maps were classically used to visually access the “key & lock” complementarity between molecules baring bioisosteric moieties and their respective receptors. The AED tool, as a computer-aided drug design resource, offers utility that extends beyond the quantitative evaluations of bioisosteric moieties.^1,4–9^ This tool is capable of clustering and matching different conformers of molecules,^10^ where conformers within a cluster share similar electrostatic potential maps. This clustering enables significant time savings as it would be sufficient to consider one representative conformer per cluster to study the “key & lock” complementarity with a given receptor.

The AED tool is based on the quantum theory of atoms in molecules (QTAIM), which is a partitioning scheme to separate atomic basins for atoms within a molecule. Calculating the AED value for a single bioisosteric moiety involves several steps.^1,4–10^ These include (i) building the molecule of interest, (ii) running a quantum simulation to obtain the wavefunction of the optimized structure, (iii) performing postprocessing on the wavefunction to obtain atomic properties, (iv) extracting volumes and electron populations for each atom, at a specific isodensity, (v) selecting the atoms relevant to the bioisosteric moiety, adding the volumes (*V*_*i*_) as well as the electron populations (*N*_*i*_) of the selected atoms, then calculating the ratio of these sums ${AED}_{bioisostere}=\frac{\sum N_{i}}{\sum V_{i}}$ where *i* is the running index for the atoms in the bioisosteric moiety). In brief, obtaining the AED for a targeted bioisosteric moiety within a drug molecule is a lengthy process involving steps that can be computationally expensive . Thus, in this study, a new approach is developed to quickly, within seconds to a few minutes, estimate accurate and reliable AED values without the need for the time-consuming quantum mechanical simulations. This approach is called “AED-Est”, which stands for “Average Electron Density Estimator”. It can be used for single atoms in molecules, groups of atoms in molecules, or the entirety of molecules.

AED-Est takes advantage of the transferability concept within the framework of QTAIM. QTAIM has a transferability principle, wherein the properties of a specific element of a given type (*i.e.*, within a particular environment) in one molecule are generally reproduced for the same element of the same type within another molecule.^11–13^ The AED tool has inheritably proven to be transferable, as the AED value for a given moiety is conserved, within a margin of *ca.* 5%, regardless of its environment.^1,4–9^ The concept of the AED-Est tool is based on having tabulated AED values (as well as volumes and electron populations) of different elements of different types within diverse environment. The objective is to be able to obtain, using this table, accurate estimates of the AED values for different atoms in molecules of various “element_at_AtomType”, without the need to engage in a lengthy process of computationally expensive quantum simulations.

As will be shown in the results and discussion, using an existing scheme for assigning atom types, such as the general Amber force field^14^ GAFF2 atom types, is not sufficiently accurate for the AED values. Therefore, a new scheme of defining atom types is developed, it is referred to as the AAA (after the author’s name, Alya A. Arabi) scheme. Although the AAA scheme was explicitly developed to rapidly estimate AED values using the AED-Est tool, it can also serve as a guide for future force-field designs, similar to how the quantum chemical topology atom types were proposed for this purpose.^15^ Similar approaches have been considered, in the literature, for carbon^16^ as well as hydrogen, oxygen, nitrogen, and sulfur,^17^ but not for phosphorus, which is included in the AAA scheme. The AAA atom types are defined by determining the element, the type of bonds (single, double, triple) or the hybridization it has, the first-degree neighboring atoms, and the second-degree neighboring atoms via covalent bonds, or even noncovalent bonds as needed. More details about the AAA schemes are provided in the Methodology Section. Ideally one needs to find the balance between accuracy and feasibility as well as transferability.^18,19^ Accuracy can be achieved by having more specific atom types, and feasibility is achieved by having fewer atom types.

In order to create the table of atomic properties (including volumes, electron populations, and AED values) per element of a given type, a data set of molecules with a wide variety of atom types is needed. Toward this objective, a data set of 553 neutral molecules is constructed in this study (for more details, please refer to the “*Training Set: 553 Molecules Grouped in Multiple Sets*” part of the “Methodology” section). This data set contains elements that account for up to 99% of the elemental composition of drug molecules. A subset of 462 molecules was systematically built from the following elements: H, C, N, O, P, and S. Each molecule has a central heavy atom (C, N, O, P, or S) bonded, via all possible bond types (single, double, or triple) to all feasible combinations of the six elements. The other subset of molecules was built by capping 91 (confirmed or potential) biosisosteric moieties with a methyl group. This built data sets was used as a training set to obtain the reference atomic properties of a large variety of atom types. In addition, another data set of 101 neutral molecules (also composed of confirmed or potential biosisosteric moieties capped with a methyl group) was created as a test set to validate the accuracy of the predictions using the reference values obtained from the training set. These two data sets can serve as benchmark data sets for evaluating the accuracy of any parametrized method to predict AED values, or even atomic properties such as volumes. In addition, the atomic properties listed for these data sets can be useful as atomic-level features in machine learning and artificial intelligence models as suggested in refs 20 and 21 .

Overall, the aim of this paper is to find a method for a rapid estimation of atomic properties at the accuracy of quantum methods. The focus is particularly on the AED values, provided their relevance to drug design.^1,4–10^ This method involves the development of a new of scheme, the AAA scheme, for defining atom types. It also involves building training and test sets of molecules that contain *i)* a wide variety of atom types, and *ii)* potential or confirmed bioisosteric moieties to link the accuracies in predicting AED values with their biological implications and drug design. Lastly, three different training sets/subsets of molecules are used to demonstrate the high level of transferability and accuracy of the method, irrespective of the training set.

## Methodology

### The AAA Scheme for Defining Atom Types

As will be shown in the “Results and Discussion” Section, using an existing scheme of defining atom types, such as the AMBER GAFF2 atom types, is unlikely to result in precise predictions of the AED values. Thus, a new scheme, referred to as the AAA scheme, is proposed to systematically define atom types. In this scheme, each atom is defined by “element_at_AtomType” where *element* is the identity of the atom of interest, and the *atom type* includes data about the neighboring atoms and the types of bonds (or hybridization) connecting them to the atom of interest. The bonds can be single (symbolized by “s”), double (symbolized by “d”), or triple (symbolized by “t”) bonds. In a given environment, priority is given sequentially for triple, double, then single bonds. For example, the carbon atom in hydrogen cyanide would be C_at_tNsH, where the triple bond to N is reported before the single bond to H. In addition to the bond type, the identity of the neighboring atoms (*i.e.*, atoms that are covalently bound to the atom of interest) is specified using the element symbols: H for hydrogen, C for carbon, O for oxygen, N for nitrogen, P for phosphorus, S for sulfur, F for fluorine, Cl for chlorine, and Br for bromine. If the atom of interest is connected via multiple bonds of the same order, *e.g.*, four single bonds, the neighboring atoms will be listed alphabetically. For example, the carbon atom in methanol would be assigned the following atom type: C_at_sHsHsHsO, and the atom type of the carbonyl C in ethanamide would be C_at_dOsCsN. If including only the first-degree neighboring atoms is deemed insufficient, second-degree neighboring atoms can also be included in the atom type. The second-degree neighboring atoms are those connected to each of the first-degree neighboring atoms. In this study, covalently bound second-degree atoms were considered only when assigning atom types of hydrogen atoms. For example, the hydroxyl H in methanol would be assigned the following atom type: H_at_O_at_sHsC. According the the AAA scheme, if including covalently bound second-degree neighboring atoms was also deemed insufficient, then noncovalently bound atoms that are within the proximity of the atom of interest can also be included. This would be highly valuable in cases involving conformational changes, such as those occuring during geometry optimization, which can affect the atomic properties even when the first- and second-degree neighboring atoms remain unchanged. Figure 1 below is a schematic diagram summarizing the AAA scheme of assigning atom types.

**Figure 1:**
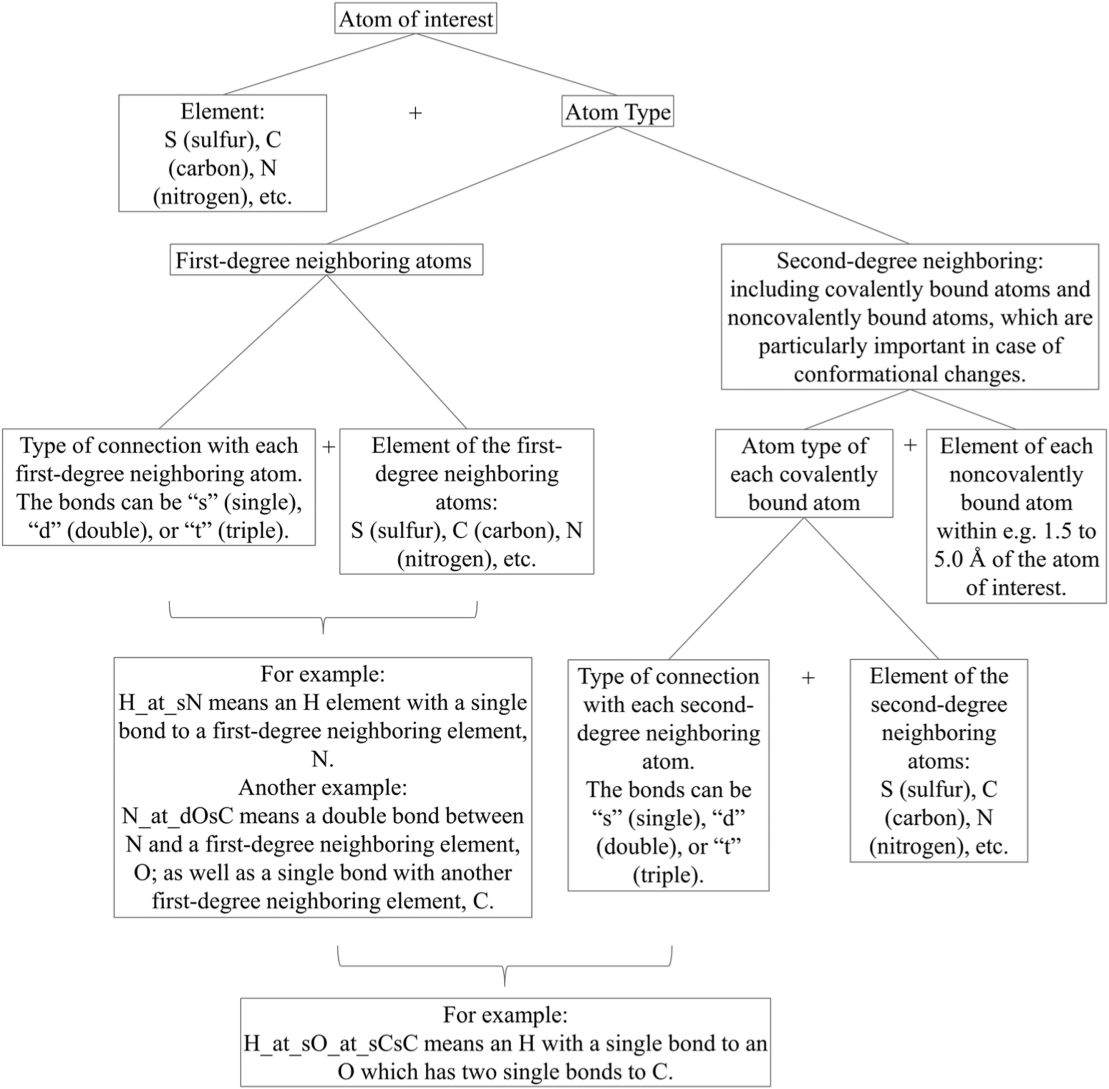
A diagram illustrating the AAA scheme for defining atom types.

### AAA vs GAFF2 Atom Types

The AMBER^14^ GAFF2 scheme assigns atom types based on element identity (*e.g.*, C, N, O, etc.), hybridization state (*e.g.*, sp3, sp2, sp), aromaticity, bonding environment (number and type of bonded atoms, ring membership such as aromatic rings, presence of electron-withdrawing/donating groups), and formal charge (where applicable). For example: c3 represents an sp3 carbon, oh represents a hydroxyl oxygen, na represents an aromatic nitrogen, etc.

To compare the precision and accuracy in predicting AED values using atom types assigned by the AAA vs GAFF2 schemes, the study was conducted separately for each scheme. The AAA atom types were assigned using the AAA scheme shown in Figure 1 (only covalently bound first- and second-degree neighboring atoms were included in the analysis; the incorporation of noncovalently bonded neighbors is proposed as a potential extension but was not investigated in the present work), and the GAFF2 atom types were assigned using antechamber in ambertools22.^22^ In the test set, three of the nitrogen atoms were assigned as a GAFF2 atom type of nu, they were manually fixed to n.

### Training Set: 553 Molecules Grouped in Multiple Sets

A new training data set of 553 molecules with a total of 4908 atoms was prepared. It is built using two subsets of organic molecules composed of H, C, N, O, P, S, F, Cl, and Br. One subset consists of 462 systematic molecules (referred to as "*Systematic*"), and another subset consists of 91 primarily cyclic (both cyclic aliphatic and cyclic aromatic) and some aliphatic molecules, referred to as "*D-Molecules*", which stands for “Diverse” molecules (see Figure 2). The subset of 462 systematic molecules are built by systematically combining six elements: H, C, N, O, S, and P. The atoms are connected with all possible types of covalent bonds, including single, double, and triple. For more information on the molecules, please refer to Table 1.

**Table 1:**
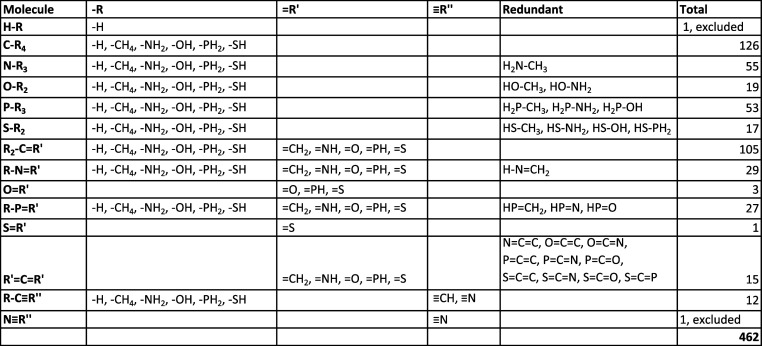
List of the Systematic Molecules Used in the Training, and Their Count. R_2_, R_3_, and R_4_ Are Not Limited to an Identical R Group Repeated 2, 3, Or 4 Times; They Can Be Any Combination of the Listed −R groups. The “Redundant” Column Lists the Molecules That Have Been Already Counted in Previous Rows and Shall Be Excluded to Avoid Double Counting

**Figure 2:**
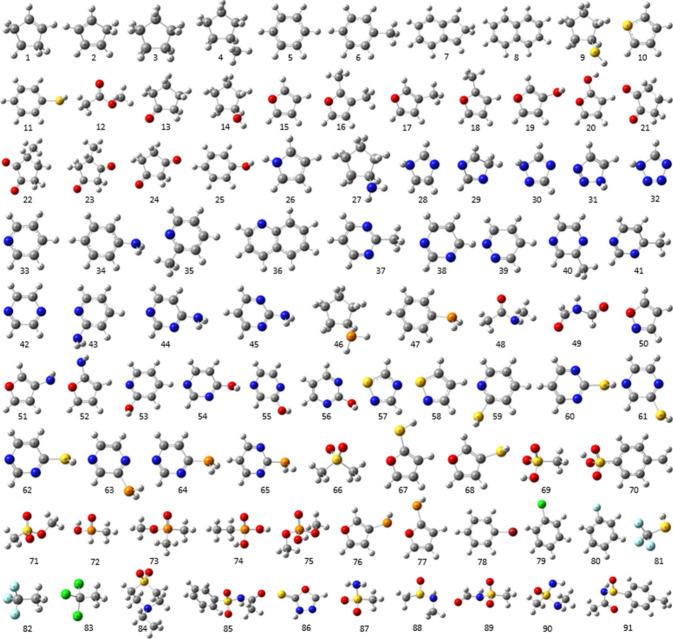
Ball-and-stick models of the 91, primarily cyclic with some aliphatic, molecules in the training set to train the AED-Est tool.

In this study, the AED-Est tool was trained on three different sets/subsets of molecules. These include *i)* all 553 molecules (referred to as *All*), *ii)* 91 primarily cyclic and some aliphatic molecules (*D-Molecules*), *and iii)* 462 systematic molecules (*Systematic*). The type of each atom in each of the 553 molecules was assigned using the AAA scheme.

### Computational Details

All molecules considered in this study we optimized using a level of theory denoted by B3LYP/6-311++G­(d,p)//B3LYP/6-311++G­(d,p). The Gaussian 16 package^23^ was used for the optimizations and for obtaining the wavefunctions. These wavefunctions were subsequently analyzed using the AIMAll^24^ software, which evaluates the atomic properties according to QTAIM.^25–27^ Atomic basins are delimited by zero-flux surfaces and by an outer isodensity envelope of 0.001 atomic unit (au). Atomic properties including electron populations, volumes, and AED values were evaluated for each atom in each molecule by averaging the corresponding operator over each atomic basin.

### Statistical Details

The mean, x̅ , of the volumes or electron populations over all atoms, in a given set, sharing the same element and atom type is given by

$$\mathrm{x̅}=\frac{1}{n}\overset{n}{\underset{i=1}{\sum }}x_{i}$$

where *n* is the total number of atoms sharing the same element and atom type, and $x_{i}$ is the individual value per atom.

The standard deviation (SD), or σ, of the volumes or electron populations atoms, in a given set, sharing the same element and atom type is given by

$$\sigma =\sqrt{\frac{1}{n-1}\overset{n}{\underset{i=1}{\sum }}{\left(x_{i}-\mathrm{x̅}\right)}^{2}}$$

where *n* is the total number of atoms sharing the same atom type and $x_{i}$ is the individual value per atom.

The AED of an atom in a molecule, a group of atoms in a molecule, or an entire molecule is given by

$$AED=\frac{\sum N_{i}}{\sum V_{i}}$$

where ∑*N*_*i*_ is the sum of the electron populations, ∑*V*_*i*_ is the sum of the atomic volumes of all atoms, and *i* represents each atom of interest within a given molecule.

The standard deviation of the AED is given by

$$\sqrt{{\left(\frac{\sigma_{N}}{{\mathrm{x̅}}_{N}}\right)}^{2}+{\left(\frac{\sigma_{V}}{{\mathrm{x̅}}_{V}}\right)}^{2}}\times {\mathrm{x̅}}_{AED}$$

where $\frac{\sigma_{N}}{{\mathrm{x̅}}_{N}}$ is the ratio of the standard deviation to the average of the electron populations per element at a given atom type, similarly $\frac{\sigma_{V}}{{\mathrm{x̅}}_{V}}$ is the ratio of the standard deviation to the average of the volumes per element at a given atom type, and ${\mathrm{x̅}}_{AED}$ is the average AED value per element at a given atom type.

The percent standard deviation of any property is given by

$$\%\;\sigma =\frac{\sigma }{\mathrm{x̅}}x100$$

where σ is the standard deviation of a given property, and x̅ is the average value of this property.

All these statistical values were evaluated for each element at each atom type in each of the three sets/subsets used in this study. The collected averages and statistics per set will be tabulated as reference values to be used with the AED-Est tool.

### Test Set

To test the accuracy of the predicted AED values using the AED-Est tool, a test set of 101 molecules was prepared (see Figure 2). This set includes a total of 1567 atoms. This test set consists of (confirmed or potential) bioisosteric moieties capped with a methyl group. Compared to the 91 molecules in the training set (shown in Figure 1), the structures in the test set are of comparable or even greater complexity.

In this study, the AED of each atom in this test set was evaluated using both tools: 1) the AED tool, *i.e.*, using the steps described in the “Computational Details” Section and 2) the AED-Est tool, where the AED values are estimated by fetching the tabulated reference information generated by training the AED-Est tool on the training sets/subsets. The percent difference between the simulated AED using quantum methods (AED_Q_) and the estimated AED using the AED-Est tool (AED_Est_) is given by

$$\frac{\left({AED}_{Est}-{AED}_{Q}\right)}{{AED}_{Q}}\times 100$$

The root mean square error (RMSE) between the simulated and estimated values is given by

$$\sqrt{\frac{1}{n}\overset{n}{\underset{j}{\sum }}{\left({AED}_{Est,j}-{AED}_{Q,j}\right)}^{2}}$$

where *n* is the total number of data points *j* for which the AED was calculated, and AED_Est, *j*_ and AED_Q, *j*_ are the estimated and simulated AED values for each data point *j*, respectively.

The concept of using the AED-Est tool is based on assigning the element_at_AtomType for the atom of interest in any molecule from the test set, and matching it with the same element_at_AtomType in the reference table to obtain the corresponding tabulated reference AED. This would be the value of the estimated AED. In addition to estimating the AED of individual atoms within molecules in a test set, the AED-Est tool was also used to estimate the AED values for the bioisosteric moieties, the methyl capping groups, and the entire molecule of all 101 molecules in the test set. While obtaining the estimated AED of a single atom in a molecule is straightforward, *i.e.*, it can be directly extracted from the tabulated reference values, estimating the AED for a group of atoms or the entire molecule is not. For groups within molecules or entire molecules, after matching the element_at_AtomType for each atom *i* constituting the group of interest, the estimated volumes (*V*_*i*_) and electron populations (*N*_*i*_) need to be extracted instead of the AED. Then, the AED of the group/molecule is calculated using $AED=\frac{\sum N_{i}}{\sum V_{i}}$ . In this study, the AED values were estimated for *i)* each atom in each molecule of the test set, *ii)* each bioisostere, capping methyl group, and entire molecule in the test set (in case the molecule had more than one methyl group, the one with the lower index was considered as the capping methyl group), *iii)* each element_at_AtomType using only first-degree neighboring atoms, *iv)* each element_at_AtomType using first-degree neighboring atoms for all elements, in addition to covalently bound second-degree neighboring atoms for H, and *v)* each element_at_AtomType using the AMBER GAFF2 atom types. Then, the percent difference in simulated and estimated AED values, the R^2^, and the RMSE were evaluated for each of these five categories. The R^2^ was evaluated from the equation of the best-fit line added using Excel.

## Results and Discussion

The AED-Est tool was trained on 553 molecules, 462 of which belong to the *Systematic* subset of molecules (see Table 1 for the identity and count of these molecules), and the remaining 91 molecules, which belong to the *D-Molecules* subset, are depicted in Figure 2. This training set of 553 molecules is highly diverse, it includes cyclic and aliphatic molecules, aromatic and nonaromatic rings, single and fused rings, cycloalkanes with one to four heteroatoms, molecules with all types of bonds, various groups such as phosphate and sulfate, halogens, as well as a wide diversity of substituents including CH_3_, OH, SH, NH_2_, PH_2_, CF_3_, and CCl_3_.

The 553 molecules in the training set bare a total of 4908 atoms, some of which belong to the same atom type, with a total of 507 atom types (or 656 atom types if the covalently bound second-degree neighboring atoms for the element H are considered). In order to use the AED-Est tool, it is important to build a table with reference values. The reference volumes, electron populations, and AED values are tabulated as average values (averaged over all occurences of the same element_at_AtomType), along with their standard deviations. The *All* training set of 553 molecules resulted in a total of 507 atom types for a total of nine elements: 5, 264, 87, 26, 90, 32, 1, 1, and 1 atom types for the H, C, N, O, P, S, F, Cl, and Br elements, respectively. As described in detail in the Methodology section, the atom type captures the first-degree for all elements. The only exception is for the H element, where the second-degree neighboring atoms were also considered, for a total of 154 atom types. Therefore, a total of 656 unique reference entries are reported in Table S1. Table S1 lists the tabulated reference electron populations and volumes per element_at_AtomType (*i.e.*, averaged over all occurences of the same element_at_AtomType in the training set of 553 molecules), along with their standard deviations. The reference AED values are then also listed, per element_at_AtomType, in Table S1. Given that the AED of a molecule (or a group within a molecule) is calculated as the ratio of the sum of electron populations to the sum of volumes of the atoms in the moiety of interest (which is mathematically not the same as the sum of AED values of the atoms in the moiety of interest), the reference tables list electron populations and volumes rather than only AED values. Table S1 also lists the percent standard deviation relative to the average AED values. For element_at_AtomType with only one occurrence, the standard deviation would be nonexistent (*i.e.*, zero). In Table S1, highlighted in red are the percent standard deviations (relative to the average AED) that exceed 5%. This threshold of 5% was chosen based on the upper limit deviations in the AED as a result of changing the R group of a given bioisosteric moiety in a molecule.^1,4–9^

As can be seen from Table S1, the carbon element has 264 atom types with a wide range of AED values spanning 0.0450 au (in the dSdS atom type) to 0.2128 au (in the sOsOsOsO atom type). Similarly, the volumes of the carbon atom span a wide range from 18.8 au (in the sOsOsOsO atom type) to 162.0 au (in the dPdP atom type); and so do the electron populations (3.84 au for the dOdO atom type to 7.77 au for the dPsPsP atom type). However, at a given atom type per element, the transferability in the AED is high as obvious from the small standard deviations. The majority of the 264 atom types for carbon (96%) have small percent standard deviation in AED, not exceeding 5%. Only in nine atom types of C (*i.e.*, less than 4% of the atom types) exceeded the 5% SD threshold. This means that the AED value for an element_at_AtomType across different molecules is similar. This is reflective of the transferability inherited from the QTAIM theory,^12^ provided the atom type is properly defined. It is important to note that this analysis includes all atom types, even those represented by a single occurrence with a reported standard deviation of 0.00% (see Table S1). However, if the above analysis is repeated considering only atom types with more than a single occurrence (*i.e.*, 50 atom types in case of the carbon element), the statistics change: 18% of carbon atom types (9 out of 50) have small percent standard deviation in AEDs, not exceeding 5%, and the transferability inherited from the QTAIM theory is still valid.

In general, there is a conserved transferability in the AED of the various atom types of nitrogen, except in the N_at_dSsN, which is a rare environment. The reason for this high percent in AED deviation (22.37%) is related to the large differences in the volumes and electron populations in the aliphatic systematic molecule versus the cyclic D-Molecule that had this atom type of N.

As shown in Table S1, the AED values of the halogens, and the different atom types of phosphorus, oxygen, and sulfur, generally seem to have a good transferability, as obvious from the small ranges of the standard deviations (for the atoms with more than one occurrence). As shown in Table 2, the alerting deviations are with the hydrogen atoms that are defined with only the first-order neighboring atoms.

**Table 2:**
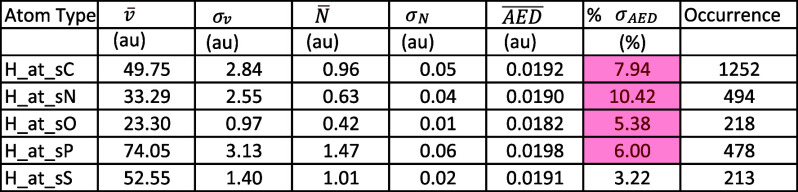
Average ± SD of the Electron Populations, Volumes, and AED Values per Atom Type of the Element H, Using the AAA Scheme with Only the First-Degree Neighboring Atoms. Highlighted in Red Are the Percent Standard Deviations (Relative to the Average AED) That Exceed 5%.

Hydrogen atoms are located on the molecular surface and often serve as primary contact points between molecules. Therefore, to accurately describe intermolecular interactions, it is important to define hydrogen atoms precisely. Table 2 clearly shows that all types of H atom, except H_at_sS, have high percent standard deviation in AED, exceeding 5%. However, it is important to note that, because of the relatively small AED values for the hydrogen atoms, every minor deviation in the absolute value can translate into a large percent deviation. To rule out the possibility that the large standard deviations are because of overly general atom type, the H atoms were redefined to include the covalently bound second-degree neighboring atoms. This resulted in a massive increase in the atom types, for the H element, from 5 to 154. Having more specific atom types for H did reduce the range of standard deviations in many of the types, except 10 of them (see Table S1). However, it is worth noting that the maximum percent standard deviation in AED reaches 14.74% (for the H_at_sN_at_sCsHsN atom type), while it did not exceed 10.5% with the five atom types including only the first-degree neighboring atoms. Thus, considering more specific atom types for the H element is not necessarily advantageous. In addition, focusing only on first-degree neighboring atoms greatly simplifies the process of assigning atom types. Therefore, it may be reasonable to consider only the 5 atom types of H, given that the absolute values are small. In fact, even the similar scheme by Popelier and Aicken considers hydrogen atoms in only seven clusters,^17^ five of which are the atom types shown in Table 2 here. The other two consider hydrogen bonds, which is equivalent to the proposed scheme of considering noncovalently bound second-neighboring atoms. Nevertheless, a percent standard deviation in AED of 10.4% for H atoms (*i.e.*, 0.0020 au) is relatively minor and is insignificant particularly when evaluating the AED of a group of atoms. This is later validated in the last section of this study, when the estimated values of a group of atoms are shown to be highly correlated with their corresponding simulated values.

To compare the performance of the results of the AAA scheme with another existing scheme, the AMBER GAFF2 atom types were assigned to all atoms, and the reference electron populations, volumes, and AED values with respect to these atom types are listed in Table 3.

**Table 3:**
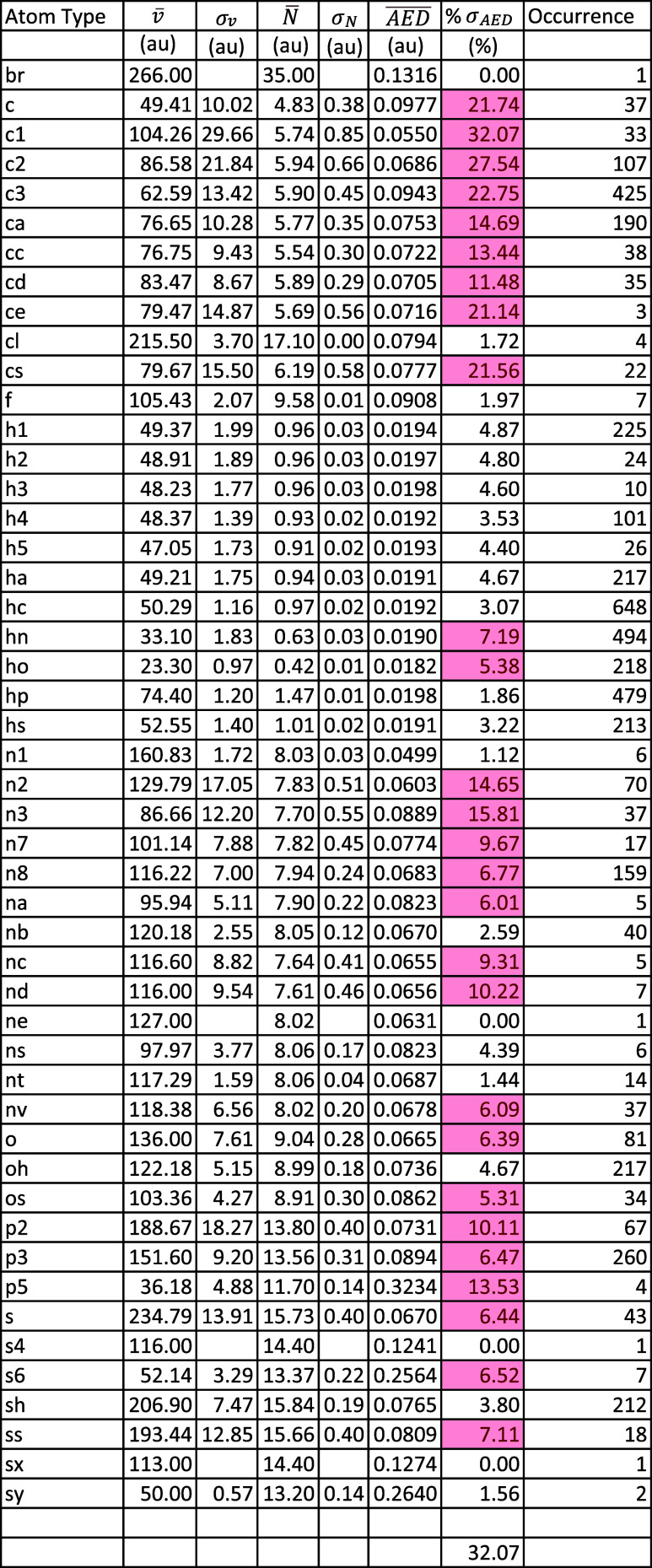
Average ± SD of the Electron Populations, Volumes, and AED Values Per Atom Type, Using the GAFF2 Atom Types. Highlighted in Red Are the Percent Standard Deviations (Relative to the Average AED) That Exceed 5%.

As shown in Table 3, the percent standard deviations with respect to the average AED values per atom type, as defined using GAFFF2, can reach up to 32.07% (see *e.g.*, the c1 atom type). However, with the atom types assigned using the AAA scheme, percent standard deviations in AED values do not exceed 22.37% (see *e.g.*, N_at_dSsN in Table S1).

More importantly, the distribution of the percent standard deviations is much better with the AAA compared to the GAFF2 atom types. Using the AAA scheme on the 553 molecules of the training set, the percent standard deviation of AED is 5–10% for only 17 out of 656 atom types (*i.e.*, 2.59% of the atom types), 10–15% for 3 atom types (*i.e.*, 0.46% of the atom types), and 15–23% for 1 atom type (*i.e.*, 0.15% of the atom types). This is a total of 3.20% of the atom types with percent standard deviations exceeding 5%. For the remaining 96.80% of atom types, the percent standard deviation of AED remains below 5%. However, using the GAFF2 atom types on the training set of 553 the molecules , the percent standard deviations is 5–10% for 13 out of 49 atom types (*i.e.*, 26.53% of the atom types), 10–15% for 7 atom types (*i.e.*, 14.29% of the atom types), and 15–23% for 5 atom types (*i.e.*, 10.20% of the atom types), in addition to 2 atom types (*i.e.*, 4.08% of the atom types) with 27.54% and 32.07% percent standard deviations in the AED. This accounts for 55.10% of the atom types with percent standard deviations exceeding 5%, while fewer than half have AED standard deviations within 5%. The reader is reminded that the reported statistics are for all atom types (including those with a single occurrence), whether for the AAA scheme or the GAFF2 scheme.

The advantage of the GAFF2 atom types is in their performance with hydrogen atom types. In this scheme, 9 out of 11 hydrogen atom types have percent standard deviation in AED below 5%, with the remaining two types staying under 10% (7.19% for hn and 5.38% for ho). In contrast, the AAA scheme, despite accounting for covalently bound second-degree neighboring atoms, yields a percent standard deviation as high as 14.74% for the H_at_sN_at_sCsHsN atom type, double the highest value observed with the GAFF2 scheme. The AAA scheme gives equivalent values to the GAFF2 when only the first-degree neighboring atoms are considered (see Table 2).

To evaluate the extent of transferability of the tabulated values, regardless of the training set/subset used, the electron populations, volumes, and AED values were evaluated separately for the 462 *Systematic* subset of molecules (see Table 1) and the subset of 91 *D-Molecules* (see Figure 2). The RMSE of the average AED between the *All-Systematic* and *All-D-Molecules* set-subset is 0.00094 au, while between the *Systematic* and *D-Molecules* subsets, it increases to 0.00133 au. The largest RMSE is only 1.8% of the smallest average for each of the three sets/subsets. In addition, Table 4 shows the values when considering each of the *Systematic* and *D-Molecules* subsets.

**Table 4:**
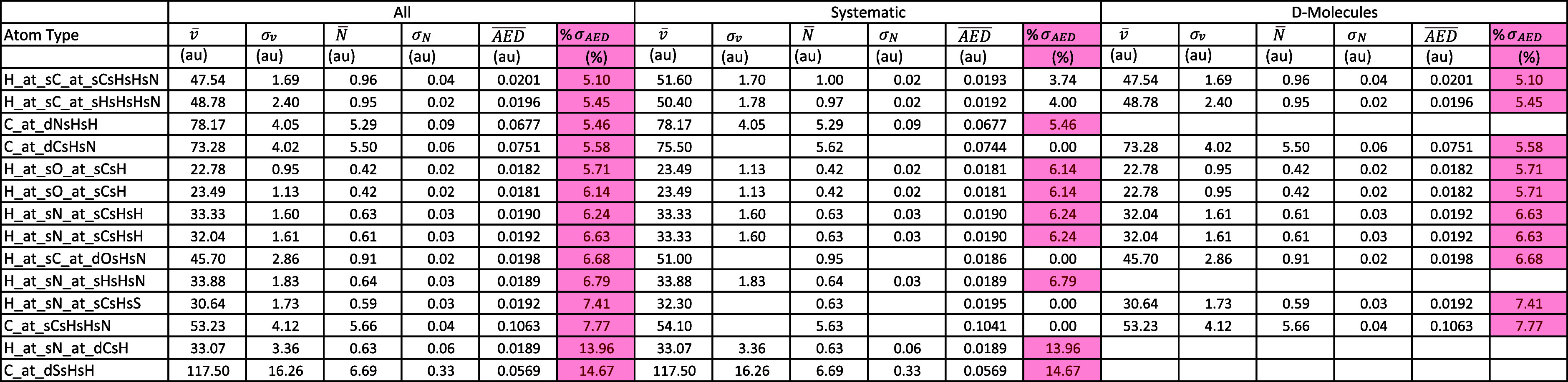
Average and Standard Deviation of the Electron Population, Volume, and AED Based on Three Different Sets/Subsets of Molecules. Only Elements at Atom Types That Have Percent Standard Deviation in AED Greater than 5% In Any of the Three Sets/Subsets Are Included. For the Hydrogen Element, Atom Types Based on First-Degree As Well As First-and-Second-Degree Neighboring Atoms Are Considered; Otherwise, For the Rest of Element, Only the Former Is Considered. The Standard Deviations of an Atom Type with One Occurrence Do Not Exist. Highlighted in Red Are the Percent Standard Deviations of the AED That Exceed 5%.

Table 4 includes only the atom types that had percent standard deviation in AED greater than 5% when the *All* set was used. It is obvious from Table 4 that changing the set/subset does not really make much of a difference in the values. Moreover, irrespective of the set/subset used, the percent standard deviation in AED did not exceed 5% for any of the atoms types except the ones reported in Table 4 and six additional types when using the subset of *D-Molecules*. These are C_at_dCsHsN (5.58%), C_at_sCsHsHsN (7.77%), H_at_sC_at_dOsHsN (6.68%), H_at_sN_at_sCsHsS (7.41%), H_at_sC_at_sCsHsHsN (5.10%), and H_at_sC_at_sHsHsHsN (5.45%). Overall, considering the RMSE values and the observations from Table 4, using different sets/subsets does not seem to make much of a difference in this case.

In the following step, to test the validity of the AED-Est tool, a test set of 101 molecules was built (see Figure 3).

**Figure 3:**
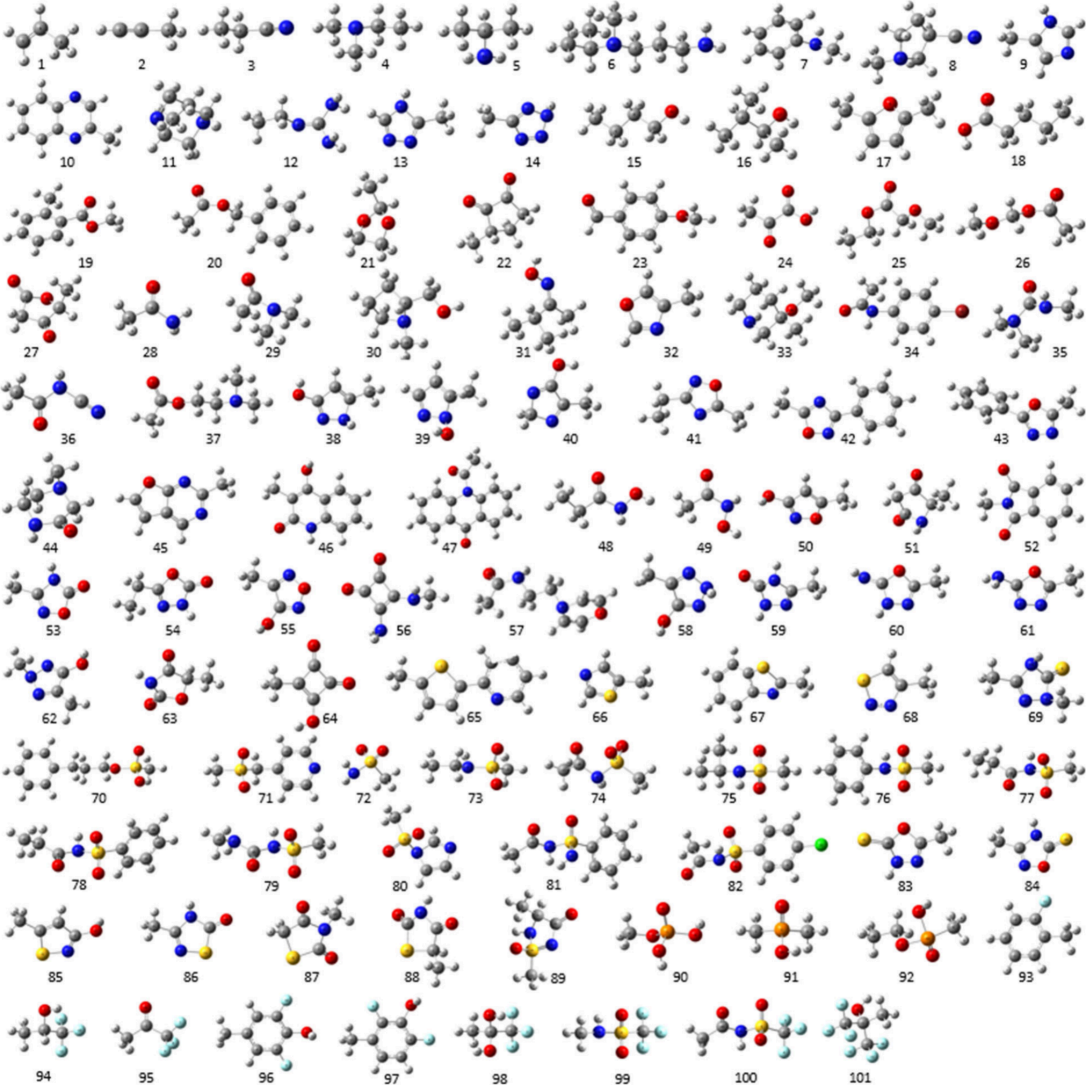
Ball-and-stick models of the 101 molecules in the test set used to validate the predictive accuracy of the AED-Est tool.

As shown in Figure 3, the test set does not share any molecules with the training set. The molecules in the test set are methyl-capped (confirmed or potential) bioisosteric moieties that are either similar in complexity to those in the training set, or even more complex. The 101 molecules in the test include a total of 1567 atoms, some of which belong to the same atom type, with a total of 142 distinct atom types (when the covalently bound second-degree neighboring atoms for the element H are considered). These include 39, 55, 23, 12, 3, 7, 1, 1, and 1 atom types for the H, C, N, O, P, S, F, Cl, and Br elements, respectively. In order to validate the AED-Est tool, the electron populations, volumes, and AED values were calculated using quantum simulations, and then estimated using the AED-Est tool. The accuracy of the AED-Est tool is then evaluated by comparing the estimated values with the simulated values. Given that the test set comprises confirmed or potential bioisosteric moieties capped with a methyl group, and considering the biological relevance of AED with respect to bioisosterism, AED values were calculated for each bioisosteric moiety, each methyl group, and the entire molecule across all 101 molecules in the test set. The estimated values were obtained by using the tabulated values using all 553 molecules in the training set. The percent differences between the estimated and the simulated values are depicted in Figure 4.

**Figure 4:**
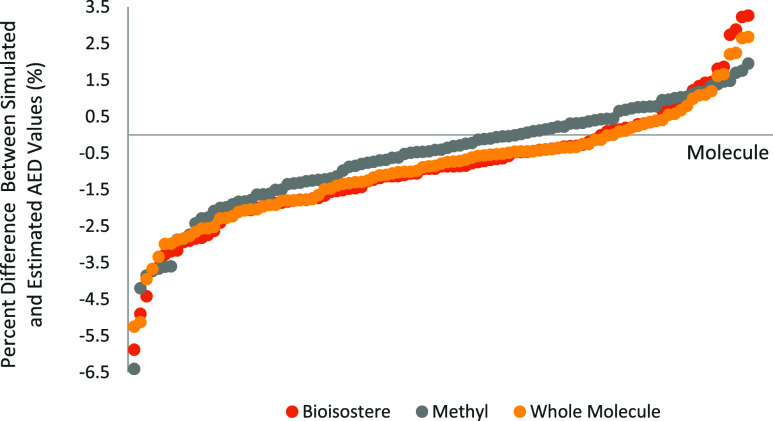
Percent difference between estimated and simulated AED values for each of the bioisosteric moiety, capping methyl group, and the entire molecule across the 101 molecules in the test set.

The graph in Figure 4 contains 303 points (101 molecules × 3), representing the bioisosteric group, the methyl group, and the entire molecule for each of the 101 molecules in the test set. This figure clearly depicts that all differences were within ±5% difference, except one methyl group (in Molecule 101), one bioisostere (in Molecule 81), and two entire molecules (Molecules 81 and 101), which had estimated AED values that are off from the simulated AED values by −5.1% to −6.4%. The prediction accuracies are best for the methyl groups, and those of the entire molecules are slightly better than those of the bioisosteres. Overall, the small differences in all cases emphasize the reliability of the AED-Est tool.

In an editorial that summarizes the parameters for validating a predictive model, the importance of reporting the root-mean-square error (RMSE) is highlighted.^28^ In this study, the accuracy of the predictions was further evaluated using the coefficient of determination (R^2^) to assess the correlation between simulated and estimated values (see Figure 5), and the RMSE to quantify deviations between estimated and simulated values. The R^2^ and RMSE were evaluated for each of the following: *i)* per atom in each molecule of the test set, *ii)* per atom type available in the test set using the AAA scheme with only first-degree neighboring atoms, *iii)* per atom type available in the test set using the AAA scheme including the second-degree neighboring atoms for the hydrogen atoms, *iv)* per GAFF2 atom type, and *v)* per 1) bioisosteric moiety, 2) methyl group, and 3) entire molecule in the test set. In all cases, the estimated values were those obtained from the averages tabulated using the training set of 553 molecules (see the “*Training Set*” part in the “Methodology” section).

**Figure 5:**
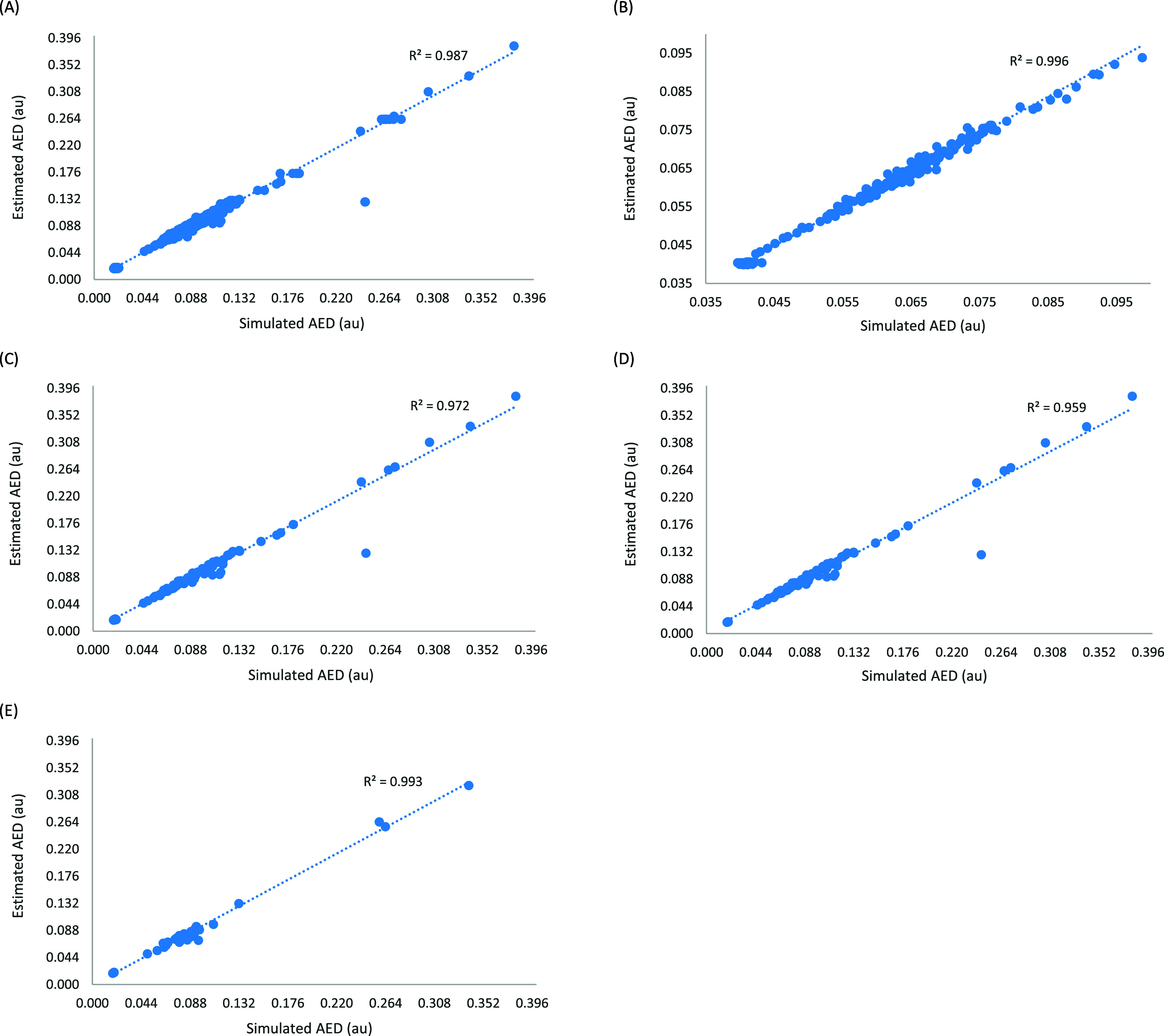
Estimated versus computed AED (A) Per atom type, (B) per bioisosteric moiety, methyl capping group, and entire molecule, (C) per AAA atom type including second-degree neighboring atoms for H, (D) per AAA atom type using only first-degree neighboring atoms, and (E) per GAFF2 atom type.

Figure 5(A) depicts the estimated vs computed AED of 1567 atoms in 101 molecules. The R^2^ of 0.987 shows that the predicted values, based on the training set of 553 molecules, are very accurate. The only outliers are for two S elements of type dNdOsCsN. The RMSE for the 1567 atoms is 4.87 × 10^–3^ au which is almost 1 order of magnitude smaller than the smallest simulated AED (0.0177 au). In addition, among the 1567 atoms in the 101 molecules of the test set, over 90% of the atoms had estimated AED values that deviated from the simulated values by less than 5%. Only 145 atoms (fewer than 10%) deviated by more than 5%, including 56 hydrogen atoms, 67 carbon atoms, 8 nitrogen atoms, 7 oxygen atoms, 4 sulfur atoms, and 3 fluorine atoms. In broader terms, 1567 atoms are within −18.71% (except for S_at_dNdOsCsN, for which the difference reached −48.37%) and 10.44%.

The R^2^ of 0.996, depicted in Figure 5(B), evidently demonstrates the high accuracy level of the AED-Est tool in predicting the AED values of parts of the molecule (bioisosteric moieties and capping methyl groups) or even the entire molecule. The accuracy in predicting groups is better than that of individual atoms. The RMSE for the bioisosteric moieties, methyl capping groups, and entire molecules are 1.35 × 10^–3^, 6.67 × 10^–4^, 1.16 × 10^–3^ au, respectively. These also are at least 1 order of magnitude smaller than the smallest computed AED values (0.0440 au, 0.0397 au, and 0.0423 au, respectively).

Figure 5(C) is a plot of the estimated vs computed AED values per atom type, including the second-degree neighbor atoms for hydrogen atoms. A total of 142 atom types were found in the 101 test molecules. The R^2^ is the least (0.972) among the R^2^ in all plots in Figure 5. However, excluding the sole outlier, an S element with the dNdOsCsN atom type, increases the R^2^ value to 0.996. The RMSE is 1.07 × 10^–2^ au (or 3.75 × 10^–3^ au, if the one outlier of S_at_dNdOsCsN is excluded), which is also an order of magnitude smaller than the smallest computed AED (0.0183 au).

Similarly, in Figure 5(D) (for 107 atom types using the AAA scheme with only first-degree neighboring atoms), the R^2^ increases from 0.959 to 0.995 if this same outlier of S_at_dNdOsCsN is excluded. The RMSE is 1.23 × 10^–2^ au (or 4.31 × 10^–3^ au, if the one outlier of S_at_dNdOsCsN is excluded), which is also an order of magnitude smaller than the smallest computed AED (0.0185 au).

Lastly, as shown in Figure 5(E), the R^2^ for 42 GAFF2 atom types is 0.993 Figure 5. The RMSE is 6.52 × 10^–3^ au, which is also an order of magnitude smaller than the smallest computed AED (0.0185 au). Although the GAFF2 atom types resulted in highly scattered AED values (as obvious from the large percent standard deviations in Table 3), the accuracy of the predicted variables is comparable with those of the AAA scheme.

Gramatica and Sangion point out the criteria for a high predictive capacity.^28^ They refer to Golbraikh and Tropsha^29^ to state that, with an external set (*i.e.*, the test set in this study), the R^2^ of the predicted (referred to here as estimated) and the experimental (referred to here as simulated) should be close to unity, and the slope has to be close to 1, meaning that the line should cross the origin without constraints. All three criteria are adequately met in all graphs shown in Figure 5, and overall, the minimal differences between the values obtained from the AED-Est tool and the quantum simulations confirm the accuracy and reliability of the newly introduced AED-Est tool.

## Limitations and Future Work

There are small differences of up to 0.5% in calculating the AED by *e.g.*, dividing N/V (using Excel, Python, and/or bash scripting) vs extracting the reported N/V in the AIMAll file. Also, despite the large number of molecules used in the training set, some atom types occurred only once. Therefore, in the future, larger data sets could be used to report revised reference values. The AED values depend on conformational changes,^10^ therefore it would be necessary to include the noncovalently bound second-degree neighbors, as needed. Future research could explore whether, for molecules or molecular groups with resonance (including aromaticity), the AED is best represented by an average across all structures or whether the AED of a single structure is acceptable. Given that the AED tool had been proven in previous studies to be applicable even for ionic molecules, reference tables for anionic and cationic molecules are currently being built and analyzed.

## Conclusions

The AED-Est tool is introduced as an accurate, precise, and rapid tool to evaluate the AED values of atoms in molecules, groups of atoms, or entire molecules. This tool eliminates the need for computationally expensive quantum simulations to evaluate AED values. A new scheme of assigning atom types, the AAA scheme, is also introduced. The AAA scheme can also serve as a guide for future force-field designs. Two data sets, that can be used as benchmark sets, are built. One was used to train the AED-Est tool, and the other was used to test the accuracy of the AED predictions by this tool. The AED-Est was trained on three separate sets/subsets: the *All* set of 553 molecules, the *Systematic* subset of 462 molecules and the *D-Molecules* subset consisting of a mixture of 91 mostly cyclic with some aliphatic molecules. Using the AAA scheme on the *All* training set of 553 neutral molecules, reference electron populations, volumes, and AED values of 507 (or 656, if the second-degree neighboring atoms are considered for H) unique atom types, according the the AAA scheme, were reported. The reference values were reported as averages over all occurences per atom type, along with their standard deviations. Using the GAFF2 atom type, the atomic properties and standard deviations of a total of 49 unique atom types were tabulated. Considering all atom types, irrespective of their occurrences, the percent standard deviations of 96.8% of the AAA atom types are less than 5%. While only 3.2% of the AAA atom types have percent standard deviations in AED greater than 5% (not exceeding 15%, except for one sulfur type at 23%), as many as 55.1% of the GAFF2 atom types have percent standard deviations exceeding 5%, with values reaching as high as 32%. The R^2^ between the simulated and estimated AED values *i)* per atom type, *ii)* per bioisosteric moiety, methyl capping group, and entire molecule, *iii)* per AAA atom type including second-degree neighbors for H, *iv)* per AAA atom type using only first-degree neighboring atoms, and *v)* per GAFF2 atom type, exceeds 0.95, and the RMSE was often at least an order of magnitude smaller than the smallest computed AED. All this data shows that the AED-Est tool results in highly accurate and precise predictions, and that using the AAA scheme for assigning atom types boosts the performance of this tool compared to using the GAFF2 atom types. The AED-Est tool has a high transferability among three different sets/subsets used (*All, Systematic*, and *D-Molecules*), as the largest RMSE relative to the smallest averages across the three sets/subsets was only 1.8%.

Overall, using the AED-Est tool with the AAA atom type assingnment scheme, can be reliably used to rapidly predict accurate and precise AED values of atoms in molecules, groups of atoms such as bioisosteric moieties, or entire molecules. The AED-Est tool and the AAA scheme can be useful in drug design. They can also be integrated into existing molecular modeling frameworkst advance computational chemistry and related fields that benefit from innovations in computer-aided predictions, including machine learning and artificial intelligence.

## Supplementary Material



## Notes

Table S1 includes the individual electron populations, volumes, and average electron densities, per atom type, along with their standard deviations, and the percent standard deviations. The data is based on the *All* set of 553 molecules (PDF)
