# Supplementary material for: Rapid and Accurate Estimation of Quantum Average Electron Densities Using Newly-Defined Atom Types
Source: ACS Omega. 2025 Aug 15;10(33):37462–73. doi: 10.1021/acsomega.5c03444 (PMC12391957; doi:10.1021/acsomega.5c03444)
Supplement: Supplementary file 1 [file ao5c03444_si_001.pdf]

Rapid and Accurate Estimation of Quantum Average Electron Densities Using Newly-Defined Atom Types

Alya A. Arabi[1]

[1]College of Medicine and Health Sciences, Department of Biochemistry and Molecular Biology, United Arab Emirates University, Al Ain, P. O. Box: 15551, United Arab Emirates.

*Corresponding author: alya.arabi@uaeu.ac.ae; alya.arabi@dal.ca

Supplementary Information

Table S1: Average ± SD of the electron populations, volumes, and AED values per atom type, using the AAA scheme with second-dergee neighboring atoms for the element H. Highlighted in red are the percent standard deviation (relative to the average AED) that exceed 5%.

Element_at_AtomType	$\bar{v}$ (au)	σ_v (au)	$\bar{N}$ (au)	σ_N (au)	$\overline{AED}$ (au)	σ_{AED} (au)	% σ_{AED} (%)	Occurrence
Br_at_sC	266.00		35.00		0.1316	0.00	0.00	1
C_at_dCdC	115.00		6.32		0.0550	0.00	0.00	1
C_at_dCdN	86.40		5.44		0.0630	0.00	0.00	1
C_at_dCdO	76.50		5.17		0.0676	0.00	0.00	1
C_at_dCdP	138.00		6.93		0.0502	0.00	0.00	1
C_at_dCdS	119.00		6.74		0.0566	0.00	0.00	1
C_at_dCsBrsC	75.60		6.08		0.0804	0.00	0.00	1
C_at_dCsCsC	71.55	0.85	5.99	0.01	0.0837	0.00	1.21	11
C_at_dCsCsCl	71.80		5.95		0.0829	0.00	0.00	1
C_at_dCsCsF	63.70		5.54		0.0870	0.00	0.00	1
C_at_dCsCsH	85.26	2.19	6.00	0.01	0.0703	0.00	2.58	138
C_at_dCsCsN	62.80	1.12	5.60	0.04	0.0892	0.00	1.89	4
C_at_dCsCsO	63.64	1.77	5.53	0.04	0.0869	0.00	2.90	5
C_at_dCsCsP	87.07	1.59	6.51	0.01	0.0747	0.00	1.83	3
C_at_dCsCsS	75.07	1.26	6.13	0.02	0.0816	0.00	1.71	6
C_at_dCsHsH	101.08	2.61	5.98	0.05	0.0592	0.00	2.70	28
C_at_dCsHsN	73.35	3.97	5.51	0.06	0.0751	0.00	5.53	33
C_at_dCsHsO	79.79	1.84	5.55	0.03	0.0696	0.00	2.35	20
C_at_dCsHsP	101.00		6.52		0.0646	0.00	0.00	1
C_at_dCsHsS	91.73	1.96	6.17	0.03	0.0673	0.00	2.19	4
C_at_dCsNsN	56.40		5.28		0.0936	0.00	0.00	1
C_at_dCsNsO	55.65	0.92	5.15	0.03	0.0925	0.00	1.74	2
C_at_dCsNsP	77.60		6.13		0.0790	0.00	0.00	1
C_at_dCsNsS	67.50		5.75		0.0852	0.00	0.00	1
C_at_dCsOsO	53.60	0.57	4.99	0.04	0.0931	0.00	1.36	2
C_at_dCsOsP	79.50	3.54	6.06	0.05	0.0762	0.00	4.52	2
C_at_dCsOsS	68.15	2.33	5.67	0.04	0.0831	0.00	3.48	2
C_at_dCsPsP	99.00		7.01		0.0708	0.00	0.00	1
C_at_dCsPsS	88.90		6.62		0.0745	0.00	0.00	1
C_at_dCsSsS	78.80		6.24		0.0792	0.00	0.00	1
C_at_dNdN	59.10		4.47		0.0756	0.00	0.00	1
C_at_dNdO	49.10		4.15		0.0845	0.00	0.00	1
C_at_dNdP	95.70		5.61		0.0586	0.00	0.00	1
C_at_dNdS	88.10		5.63		0.0639	0.00	0.00	1
C_at_dNsCsC	58.83	2.35	5.44	0.09	0.0924	0.00	4.34	4
C_at_dNsCsH	70.83	1.58	5.45	0.05	0.0769	0.00	2.39	20
C_at_dNsCsN	49.60	1.46	5.01	0.08	0.1011	0.00	3.32	4
C_at_dNsCsO	48.43	1.66	4.90	0.08	0.1011	0.00	3.83	4
C_at_dNsCsP	72.47	3.31	5.90	0.12	0.0814	0.00	5.02	3
C_at_dNsCsS	62.05	2.22	5.54	0.11	0.0892	0.00	4.11	4

C_at_dNsHsH	78.17	4.05	5.29	0.09	0.0677	0.00	5.46	6
C_at_dNsHsN	61.02	3.11	4.99	0.05	0.0817	0.00	5.20	13
C_at_dNsHsO	58.25	3.75	4.81	0.08	0.0825	0.01	6.63	2
C_at_dNsHsP	80.60		5.77		0.0716	0.00	0.00	1
C_at_dNsHsS	73.00	5.09	5.47	0.15	0.0749	0.01	7.48	2
C_at_dNsNsN	40.40	0.28	4.58	0.03	0.1134	0.00	0.93	2
C_at_dNsNsO	38.65	0.35	4.42	0.03	0.1144	0.00	1.12	2
C_at_dNsNsP	61.70	2.26	5.41	0.06	0.0877	0.00	3.81	2
C_at_dNsNsS	51.65	1.48	5.03	0.10	0.0974	0.00	3.48	2
C_at_dNsOsO	35.40		4.20		0.1186	0.00	0.00	1
C_at_dNsOsP	59.30		5.21		0.0879	0.00	0.00	1
C_at_dNsOsS	49.10		4.83		0.0984	0.00	0.00	1
C_at_dNsPsP	81.40		6.19		0.0760	0.00	0.00	1
C_at_dNsPsS	71.80		5.82		0.0811	0.00	0.00	1
C_at_dNsSsS	62.10		5.46		0.0879	0.00	0.00	1
C_at_dOdO	40.60		3.84		0.0946	0.00	0.00	1
C_at_dOdP	93.60		5.49		0.0587	0.00	0.00	1
C_at_dOdS	82.10		5.42		0.0660	0.00	0.00	1
C_at_dOsCsC	48.97	1.18	5.00	0.01	0.1022	0.00	2.42	10
C_at_dOsCsH	57.40		4.99		0.0869	0.00	0.00	1
C_at_dOsCsN	40.90	0.61	4.61	0.03	0.1127	0.00	1.62	4
C_at_dOsCsO	39.50	0.00	4.49	0.00	0.1137	0.00	0.00	2
C_at_dOsCsP	59.50		5.29		0.0889	0.00	0.00	1
C_at_dOsCsS	52.30		5.06		0.0967	0.00	0.00	1
C_at_dOsHsH	67.10		4.98		0.0742	0.00	0.00	1
C_at_dOsHsN	50.08	1.17	4.61	0.04	0.0920	0.00	2.46	4
C_at_dOsHsO	47.80		4.43		0.0927	0.00	0.00	1
C_at_dOsHsP	70.20		5.35		0.0762	0.00	0.00	1
C_at_dOsHsS	62.00		5.05		0.0815	0.00	0.00	1
C_at_dOsNsN	33.20		4.22		0.1271	0.00	0.00	1
C_at_dOsNsO	31.20		4.06		0.1301	0.00	0.00	1
C_at_dOsNsP	53.10		4.98		0.0938	0.00	0.00	1
C_at_dOsNsS	43.30		4.61		0.1065	0.00	0.00	1
C_at_dOsOsO	28.40		3.86		0.1359	0.00	0.00	1
C_at_dOsOsP	52.80		4.91		0.0930	0.00	0.00	1
C_at_dOsOsS	42.50		4.53		0.1066	0.00	0.00	1
C_at_dOsPsP	73.60		5.74		0.0780	0.00	0.00	1
C_at_dOsPsS	65.30		5.47		0.0838	0.00	0.00	1
C_at_dOsSsS	56.00		5.14		0.0918	0.00	0.00	1
C_at_dPdP	162.00		7.64		0.0472	0.00	0.00	1
C_at_dPdS	138.00		7.29		0.0528	0.00	0.00	1
C_at_dPsCsC	95.20		6.76		0.0710	0.00	0.00	1
C_at_dPsCsH	110.00		6.80		0.0618	0.00	0.00	1
C_at_dPsCsN	81.40		6.19		0.0760	0.00	0.00	1
C_at_dPsCsO	84.30		6.16		0.0731	0.00	0.00	1
C_at_dPsCsP	110.00		7.28		0.0662	0.00	0.00	1
C_at_dPsCsS	96.80		6.83		0.0706	0.00	0.00	1

C_at_dPsHsH	130.20	2.77	6.91	0.05	0.0531	0.00	2.27	5
C_at_dPsHsN	95.20		6.20		0.0651	0.00	0.00	1
C_at_dPsHsO	97.70		6.18		0.0633	0.00	0.00	1
C_at_dPsHsP	127.00		7.34		0.0578	0.00	0.00	1
C_at_dPsHsS	112.00		6.87		0.0613	0.00	0.00	1
C_at_dPsNsN	72.50		5.74		0.0792	0.00	0.00	1
C_at_dPsNsO	73.60		5.67		0.0770	0.00	0.00	1
C_at_dPsNsP	96.00		6.67		0.0695	0.00	0.00	1
C_at_dPsNsS	85.30		6.30		0.0739	0.00	0.00	1
C_at_dPsOsO	71.00		5.48		0.0772	0.00	0.00	1
C_at_dPsOsP	98.60		6.66		0.0675	0.00	0.00	1
C_at_dPsOsS	87.10		6.23		0.0715	0.00	0.00	1
C_at_dPsPsP	121.00		7.77		0.0642	0.00	0.00	1
C_at_dPsPsS	109.00		7.30		0.0670	0.00	0.00	1
C_at_dPsSsS	98.90		6.91		0.0699	0.00	0.00	1
C_at_dSdS	137.00		6.16		0.0450	0.00	0.00	1
C_at_dSsCsC	78.00		6.40		0.0821	0.00	0.00	1
C_at_dSsCsH	90.90		6.43		0.0707	0.00	0.00	1
C_at_dSsCsN	66.00		5.84		0.0885	0.00	0.00	1
C_at_dSsCsO	68.40		5.85		0.0855	0.00	0.00	1
C_at_dSsCsP	91.30		6.89		0.0755	0.00	0.00	1
C_at_dSsCsS	80.80		6.49		0.0803	0.00	0.00	1
C_at_dSsHsH	117.50	16.26	6.69	0.33	0.0569	0.01	14.67	2
C_at_dSsHsN	78.20		5.86		0.0749	0.00	0.00	1
C_at_dSsHsO	80.40		5.87		0.0730	0.00	0.00	1
C_at_dSsHsP	106.00		6.91		0.0652	0.00	0.00	1
C_at_dSsHsS	94.30		6.53		0.0692	0.00	0.00	1
C_at_dSsNsN	56.80		5.39		0.0949	0.00	0.00	1
C_at_dSsNsO	59.80	3.11	5.39	0.07	0.0901	0.00	5.37	2
C_at_dSsNsP	79.10		6.30		0.0796	0.00	0.00	1
C_at_dSsNsS	69.20		5.95		0.0860	0.00	0.00	1
C_at_dSsOsO	56.50		5.21		0.0922	0.00	0.00	1
C_at_dSsOsP	80.50		6.29		0.0781	0.00	0.00	1
C_at_dSsOsS	70.00		5.92		0.0846	0.00	0.00	1
C_at_dSsPsP	104.00		7.36		0.0708	0.00	0.00	1
C_at_dSsPsS	93.60		6.95		0.0743	0.00	0.00	1
C_at_dSsSsS	83.10		6.58		0.0792	0.00	0.00	1
C_at_sCsClsClsCl	45.10		5.73		0.1271	0.00	0.00	1
C_at_sCsCsCsC	42.20		5.90		0.1398	0.00	0.00	1
C_at_sCsCsCsH	51.03	1.82	5.95	0.04	0.1166	0.00	3.62	4
C_at_sCsCsCsN	38.50		5.62		0.1460	0.00	0.00	1
C_at_sCsCsCsO	37.60		5.51		0.1465	0.00	0.00	1
C_at_sCsCsCsP	51.30		6.30		0.1228	0.00	0.00	1
C_at_sCsCsCsS	45.50		6.00		0.1319	0.00	0.00	1
C_at_sCsCsHsH	61.53	2.12	5.97	0.03	0.0970	0.00	3.48	46
C_at_sCsCsHsN	45.30	0.14	5.63	0.01	0.1243	0.00	0.40	2
C_at_sCsCsHsO	44.45	0.49	5.51	0.00	0.1240	0.00	1.11	2

C_at_sCsCsHsP	60.85	0.78	6.38	0.05	0.1048	0.00	1.50	2
C_at_sCsCsHsS	53.35	0.21	6.02	0.01	0.1128	0.00	0.46	2
C_at_sCsCsNsN	34.50		5.34		0.1548	0.00	0.00	1
C_at_sCsCsNsO	33.40		5.20		0.1557	0.00	0.00	1
C_at_sCsCsNsP	47.50		6.02		0.1267	0.00	0.00	1
C_at_sCsCsNsS	41.20		5.70		0.1383	0.00	0.00	1
C_at_sCsCsOsO	32.20		5.05		0.1568	0.00	0.00	1
C_at_sCsCsOsP	47.00		5.93		0.1262	0.00	0.00	1
C_at_sCsCsOsS	40.10		5.57		0.1389	0.00	0.00	1
C_at_sCsCsPsP	61.30		6.74		0.1100	0.00	0.00	1
C_at_sCsCsPsS	55.30		6.42		0.1161	0.00	0.00	1
C_at_sCsCsSsS	49.00		6.09		0.1243	0.00	0.00	1
C_at_sCsFsFsF	25.20		4.39		0.1742	0.00	0.00	1
C_at_sCsHsHsH	68.35	2.29	5.96	0.03	0.0872	0.00	3.39	144
C_at_sCsHsHsN	53.40	3.59	5.65	0.04	0.1058	0.01	6.75	5
C_at_sCsHsHsO	52.90		5.50		0.1040	0.00	0.00	1
C_at_sCsHsHsP	72.35	0.07	6.50	0.14	0.0898	0.00	2.18	2
C_at_sCsHsHsS	62.93	0.67	6.08	0.05	0.0966	0.00	1.36	3
C_at_sCsHsNsN	41.10		5.33		0.1297	0.00	0.00	1
C_at_sCsHsNsO	39.90		5.18		0.1298	0.00	0.00	1
C_at_sCsHsNsP	55.90		6.03		0.1079	0.00	0.00	1
C_at_sCsHsNsS	49.10		5.69		0.1159	0.00	0.00	1
C_at_sCsHsOsO	38.50		5.02		0.1304	0.00	0.00	1
C_at_sCsHsOsP	55.40		5.93		0.1070	0.00	0.00	1
C_at_sCsHsOsS	48.00		5.55		0.1156	0.00	0.00	1
C_at_sCsHsPsP	72.20		6.81		0.0943	0.00	0.00	1
C_at_sCsHsPsS	64.90		6.46		0.0995	0.00	0.00	1
C_at_sCsHsSsS	57.90		6.10		0.1054	0.00	0.00	1
C_at_sCsNsNsN	30.50		5.05		0.1656	0.00	0.00	1
C_at_sCsNsNsO	29.60		4.89		0.1652	0.00	0.00	1
C_at_sCsNsNsP	43.30		5.71		0.1319	0.00	0.00	1
C_at_sCsNsNsS	37.00		5.39		0.1457	0.00	0.00	1
C_at_sCsNsOsO	27.70		4.74		0.1711	0.00	0.00	1
C_at_sCsNsOsP	42.80		5.60		0.1308	0.00	0.00	1
C_at_sCsNsOsS	35.70		5.22		0.1462	0.00	0.00	1
C_at_sCsNsPsP	57.30		6.45		0.1126	0.00	0.00	1
C_at_sCsNsPsS	51.20		6.12		0.1195	0.00	0.00	1
C_at_sCsNsSsS	44.40		5.74		0.1293	0.00	0.00	1
C_at_sCsOsOsO	26.00		4.56		0.1754	0.00	0.00	1
C_at_sCsOsOsP	41.40		5.43		0.1312	0.00	0.00	1
C_at_sCsOsOsS	34.20		5.04		0.1474	0.00	0.00	1
C_at_sCsOsPsP	56.80		6.31		0.1111	0.00	0.00	1
C_at_sCsOsPsS	50.30		5.97		0.1187	0.00	0.00	1
C_at_sCsOsSsS	42.80		5.60		0.1308	0.00	0.00	1
C_at_sCsPsPsP	71.90		7.18		0.0999	0.00	0.00	1
C_at_sCsPsPsS	65.50		6.85		0.1046	0.00	0.00	1
C_at_sCsPsSsS	58.80		6.51		0.1107	0.00	0.00	1

C_at_sCsSsSsS	52.10		6.18		0.1186	0.00	0.00	1
C_at_sFsFsFsS	27.40		4.40		0.1606	0.00	0.00	1
C_at_sHsHsHsH	84.50		5.99		0.0709	0.00	0.00	1
C_at_sHsHsHsN	62.92	1.85	5.66	0.03	0.0899	0.00	2.97	35
C_at_sHsHsHsO	62.24	0.95	5.52	0.03	0.0887	0.00	1.60	12
C_at_sHsHsHsP	84.81	2.06	6.51	0.04	0.0768	0.00	2.51	36
C_at_sHsHsHsS	74.64	0.85	6.08	0.02	0.0814	0.00	1.19	15
C_at_sHsHsNsN	48.90		5.31		0.1086	0.00	0.00	1
C_at_sHsHsNsO	47.50		5.15		0.1084	0.00	0.00	1
C_at_sHsHsNsP	66.80		6.08		0.0910	0.00	0.00	1
C_at_sHsHsNsS	58.40		5.69		0.0974	0.00	0.00	1
C_at_sHsHsOsO	45.70		5.00		0.1094	0.00	0.00	1
C_at_sHsHsOsP	65.70		5.95		0.0906	0.00	0.00	1
C_at_sHsHsOsS	56.50		5.54		0.0981	0.00	0.00	1
C_at_sHsHsPsP	86.00		6.87		0.0799	0.00	0.00	1
C_at_sHsHsPsS	77.10		6.50		0.0843	0.00	0.00	1
C_at_sHsHsSsS	68.40		6.13		0.0896	0.00	0.00	1
C_at_sHsNsNsN	36.50		5.02		0.1375	0.00	0.00	1
C_at_sHsNsNsO	35.50		4.86		0.1369	0.00	0.00	1
C_at_sHsNsNsP	51.50		5.72		0.1111	0.00	0.00	1
C_at_sHsNsNsS	44.10		5.33		0.1209	0.00	0.00	1
C_at_sHsNsOsO	33.90		4.69		0.1383	0.00	0.00	1
C_at_sHsNsOsP	49.80		5.54		0.1112	0.00	0.00	1
C_at_sHsNsOsS	43.10		5.19		0.1204	0.00	0.00	1
C_at_sHsNsPsP	67.30		6.48		0.0963	0.00	0.00	1
C_at_sHsNsPsS	60.60		6.13		0.1012	0.00	0.00	1
C_at_sHsNsSsS	52.80		5.76		0.1091	0.00	0.00	1
C_at_sHsOsOsO	31.60		4.50		0.1424	0.00	0.00	1
C_at_sHsOsOsP	49.10		5.42		0.1104	0.00	0.00	1
C_at_sHsOsOsS	41.00		5.03		0.1227	0.00	0.00	1
C_at_sHsOsPsP	66.40		6.33		0.0953	0.00	0.00	1
C_at_sHsOsPsS	59.30		5.98		0.1008	0.00	0.00	1
C_at_sHsOsSsS	51.10		5.61		0.1098	0.00	0.00	1
C_at_sHsPsPsP	84.30		7.27		0.0862	0.00	0.00	1
C_at_sHsPsPsS	76.80		6.91		0.0900	0.00	0.00	1
C_at_sHsPsSsS	69.10		6.54		0.0946	0.00	0.00	1
C_at_sHsSsSsS	61.30		6.20		0.1011	0.00	0.00	1
C_at_sNsNsNsN	26.80		4.75		0.1772	0.00	0.00	1
C_at_sNsNsNsO	25.80		4.59		0.1779	0.00	0.00	1
C_at_sNsNsNsP	38.60		5.34		0.1383	0.00	0.00	1
C_at_sNsNsNsS	32.80		5.05		0.1540	0.00	0.00	1
C_at_sNsNsOsO	23.30		4.41		0.1893	0.00	0.00	1
C_at_sNsNsOsP	37.90		5.23		0.1380	0.00	0.00	1
C_at_sNsNsOsS	31.10		4.91		0.1579	0.00	0.00	1
C_at_sNsNsPsP	53.50		6.15		0.1150	0.00	0.00	1
C_at_sNsNsPsS	46.80		5.80		0.1239	0.00	0.00	1
C_at_sNsNsSsS	39.50		5.43		0.1375	0.00	0.00	1

C_at_sNsOsOsO	21.30		4.23		0.1986	0.00	0.00	1
C_at_sNsOsOsP	36.50		5.07		0.1389	0.00	0.00	1
C_at_sNsOsOsS	29.50		4.71		0.1597	0.00	0.00	1
C_at_sNsOsPsP	52.30		5.99		0.1145	0.00	0.00	1
C_at_sNsOsPsS	45.70		5.65		0.1236	0.00	0.00	1
C_at_sNsOsSsS	38.90		5.30		0.1362	0.00	0.00	1
C_at_sNsPsPsP	66.80		6.83		0.1022	0.00	0.00	1
C_at_sNsPsPsS	60.20		6.48		0.1076	0.00	0.00	1
C_at_sNsPsSsS	53.60		6.14		0.1146	0.00	0.00	1
C_at_sNsSsSsS	47.70		5.82		0.1220	0.00	0.00	1
C_at_sOsOsOsO	18.80		4.00		0.2128	0.00	0.00	1
C_at_sOsOsOsP	35.10		4.90		0.1396	0.00	0.00	1
C_at_sOsOsOsS	27.50		4.52		0.1644	0.00	0.00	1
C_at_sOsOsPsP	51.30		5.84		0.1138	0.00	0.00	1
C_at_sOsOsPsS	44.10		5.47		0.1240	0.00	0.00	1
C_at_sOsOsSsS	37.70		5.12		0.1358	0.00	0.00	1
C_at_sOsPsPsP	66.40		6.72		0.1012	0.00	0.00	1
C_at_sOsPsPsS	59.50		6.36		0.1069	0.00	0.00	1
C_at_sOsPsSsS	52.60		6.01		0.1143	0.00	0.00	1
C_at_sOsSsSsS	46.40		5.67		0.1222	0.00	0.00	1
C_at_sPsPsPsP	82.50		7.63		0.0925	0.00	0.00	1
C_at_sPsPsPsS	75.80		7.29		0.0962	0.00	0.00	1
C_at_sPsPsSsS	69.10		6.95		0.1006	0.00	0.00	1
C_at_sPsSsSsS	62.20		6.60		0.1061	0.00	0.00	1
C_at_sSsSsSsS	55.50		6.27		0.1130	0.00	0.00	1
C_at_tCsC	113.00		6.18		0.0547	0.00	0.00	1
C_at_tCsH	130.86	2.54	6.04	0.14	0.0462	0.00	2.96	7
C_at_tCsN	105.00		5.85		0.0557	0.00	0.00	1
C_at_tCsO	103.00		5.73		0.0556	0.00	0.00	1
C_at_tCsP	125.00		6.52		0.0522	0.00	0.00	1
C_at_tCsS	115.00		6.30		0.0548	0.00	0.00	1
C_at_tNsC	78.30		5.11		0.0653	0.00	0.00	1
C_at_tNsH	95.10		5.08		0.0534	0.00	0.00	1
C_at_tNsN	66.50		4.64		0.0698	0.00	0.00	1
C_at_tNsO	62.80		4.44		0.0707	0.00	0.00	1
C_at_tNsP	96.00		5.59		0.0582	0.00	0.00	1
C_at_tNsS	84.80		5.32		0.0627	0.00	0.00	1
Cl_at_sC	215.50	3.70	17.10	0.00	0.0794	0.00	1.72	4
F_at_sC	105.43	2.07	9.58	0.01	0.0908	0.00	1.97	7
H_at_sC_at_dCsCsH	49.35	1.25	0.95	0.01	0.0192	0.00	2.97	138
H_at_sC_at_dCsHsH	49.78	1.34	0.95	0.02	0.0190	0.00	3.70	56
H_at_sC_at_dCsHsN	48.37	0.52	0.93	0.01	0.0193	0.00	1.55	33
H_at_sC_at_dCsHsO	46.99	0.73	0.90	0.01	0.0193	0.00	2.10	20
H_at_sC_at_dCsHsP	49.80		0.95		0.0190	0.00	0.00	1
H_at_sC_at_dCsHsS	47.80	0.35	0.91	0.01	0.0191	0.00	1.57	4
H_at_sC_at_dNsCsH	48.32	1.00	0.93	0.01	0.0193	0.00	2.59	20
H_at_sC_at_dNsHsH	50.15	1.92	0.95	0.02	0.0189	0.00	4.48	12

H_at_sC_at_dNsHsN	47.00	0.99	0.91	0.02	0.0194	0.00	2.91	13
H_at_sC_at_dNsHsO	45.65	3.46	0.89	0.06	0.0195	0.00	9.91	2
H_at_sC_at_dNsHsP	49.10		0.94		0.0191	0.00	0.00	1
H_at_sC_at_dNsHsS	46.55	1.06	0.90	0.02	0.0193	0.00	3.51	2
H_at_sC_at_dOsCsH	52.20		0.96		0.0185	0.00	0.00	1
H_at_sC_at_dOsHsH	50.85	0.07	0.95	0.00	0.0186	0.00	0.16	2
H_at_sC_at_dOsHsN	47.03	3.53	0.92	0.03	0.0195	0.00	8.08	4
H_at_sC_at_dOsHsO	49.10		0.93		0.0189	0.00	0.00	1
H_at_sC_at_dOsHsP	51.10		0.94		0.0184	0.00	0.00	1
H_at_sC_at_dOsHsS	48.70		0.92		0.0189	0.00	0.00	1
H_at_sC_at_dPsCsH	49.40		0.94		0.0191	0.00	0.00	1
H_at_sC_at_dPsHsH	49.14	0.71	0.93	0.01	0.0189	0.00	1.85	10
H_at_sC_at_dPsHsN	48.20		0.93		0.0193	0.00	0.00	1
H_at_sC_at_dPsHsO	48.40		0.93		0.0193	0.00	0.00	1
H_at_sC_at_dPsHsP	48.30		0.92		0.0191	0.00	0.00	1
H_at_sC_at_dPsHsS	46.80		0.91		0.0195	0.00	0.00	1
H_at_sC_at_dSsCsH	48.70		0.93		0.0191	0.00	0.00	1
H_at_sC_at_dSsHsH	48.33	0.22	0.92	0.00	0.0189	0.00	0.50	4
H_at_sC_at_dSsHsN	47.90		0.92		0.0191	0.00	0.00	1
H_at_sC_at_dSsHsO	47.60		0.91		0.0192	0.00	0.00	1
H_at_sC_at_dSsHsP	47.70		0.91		0.0191	0.00	0.00	1
H_at_sC_at_dSsHsS	46.50		0.90		0.0193	0.00	0.00	1
H_at_sC_at_sCsCsCsH	50.08	1.56	0.99	0.03	0.0197	0.00	4.27	4
H_at_sC_at_sCsCsHsH	50.55	1.31	0.98	0.03	0.0193	0.00	3.65	93
H_at_sC_at_sCsCsHsN	52.40	0.14	1.02	0.00	0.0195	0.00	0.27	2
H_at_sC_at_sCsCsHsO	50.65	0.35	1.00	0.00	0.0197	0.00	0.70	2
H_at_sC_at_sCsCsHsP	50.05	0.21	0.99	0.00	0.0197	0.00	0.51	2
H_at_sC_at_sCsCsHsS	48.95	0.21	0.97	0.00	0.0198	0.00	0.57	2
H_at_sC_at_sCsHsHsH	50.34	1.16	0.97	0.02	0.0192	0.00	2.94	432
H_at_sC_at_sCsHsHsN	48.35	2.34	0.96	0.04	0.0200	0.00	6.10	10
H_at_sC_at_sCsHsHsO	50.95	0.07	0.99	0.00	0.0194	0.00	0.14	2
H_at_sC_at_sCsHsHsP	48.93	1.36	0.96	0.02	0.0196	0.00	3.47	4
H_at_sC_at_sCsHsHsS	47.62	1.19	0.94	0.02	0.0197	0.00	3.27	6
H_at_sC_at_sCsHsNsN	50.60		1.00		0.0197	0.00	0.00	1
H_at_sC_at_sCsHsNsO	51.80		1.01		0.0195	0.00	0.00	1
H_at_sC_at_sCsHsNsP	50.80		0.99		0.0195	0.00	0.00	1
H_at_sC_at_sCsHsNsS	49.70		0.98		0.0197	0.00	0.00	1
H_at_sC_at_sCsHsOsO	50.20		0.99		0.0196	0.00	0.00	1
H_at_sC_at_sCsHsOsP	49.60		0.98		0.0197	0.00	0.00	1
H_at_sC_at_sCsHsOsS	48.00		0.96		0.0199	0.00	0.00	1
H_at_sC_at_sCsHsPsP	48.70		0.96		0.0198	0.00	0.00	1
H_at_sC_at_sCsHsPsS	47.70		0.95		0.0199	0.00	0.00	1
H_at_sC_at_sCsHsSsS	46.60		0.94		0.0201	0.00	0.00	1
H_at_sC_at_sHsHsHsH	51.50	0.00	0.97	0.00	0.0189	0.00	0.00	4
H_at_sC_at_sHsHsHsN	50.26	1.88	0.97	0.02	0.0192	0.00	4.22	105
H_at_sC_at_sHsHsHsO	48.94	1.68	0.95	0.02	0.0194	0.00	3.93	36
H_at_sC_at_sHsHsHsP	49.98	0.87	0.95	0.01	0.0191	0.00	2.17	108

H_at_sC_at_sHsHsHsS	47.85	1.41	0.93	0.02	0.0194	0.00	3.67	45
H_at_sC_at_sHsHsNsN	51.50	0.00	1.00	0.00	0.0194	0.00	0.00	2
H_at_sC_at_sHsHsNsO	50.80	1.41	0.99	0.02	0.0194	0.00	3.31	2
H_at_sC_at_sHsHsNsP	50.35	1.63	0.97	0.02	0.0194	0.00	3.70	2
H_at_sC_at_sHsHsNsS	48.95	1.34	0.96	0.02	0.0196	0.00	3.19	2
H_at_sC_at_sHsHsOsO	48.00	0.00	0.95	0.00	0.0198	0.00	0.00	2
H_at_sC_at_sHsHsOsP	49.75	0.07	0.97	0.00	0.0194	0.00	0.20	2
H_at_sC_at_sHsHsOsS	47.30	1.13	0.93	0.02	0.0197	0.00	2.91	2
H_at_sC_at_sHsHsPsP	48.70	0.00	0.95	0.00	0.0195	0.00	0.00	2
H_at_sC_at_sHsHsPsS	47.85	0.07	0.94	0.00	0.0197	0.00	0.15	2
H_at_sC_at_sHsHsSsS	46.50	0.42	0.92	0.00	0.0199	0.00	0.99	2
H_at_sC_at_sHsNsNsN	49.60		0.98		0.0198	0.00	0.00	1
H_at_sC_at_sHsNsNsO	50.00		0.98		0.0197	0.00	0.00	1
H_at_sC_at_sHsNsNsP	49.60		0.98		0.0197	0.00	0.00	1
H_at_sC_at_sHsNsNsS	48.70		0.96		0.0198	0.00	0.00	1
H_at_sC_at_sHsNsOsO	50.90		0.99		0.0195	0.00	0.00	1
H_at_sC_at_sHsNsOsP	50.70		0.99		0.0195	0.00	0.00	1
H_at_sC_at_sHsNsOsS	49.00		0.97		0.0197	0.00	0.00	1
H_at_sC_at_sHsNsPsP	50.00		0.97		0.0195	0.00	0.00	1
H_at_sC_at_sHsNsPsS	48.50		0.96		0.0198	0.00	0.00	1
H_at_sC_at_sHsNsSsS	47.60		0.95		0.0199	0.00	0.00	1
H_at_sC_at_sHsOsOsO	47.00		0.93		0.0199	0.00	0.00	1
H_at_sC_at_sHsOsOsP	49.20		0.97		0.0197	0.00	0.00	1
H_at_sC_at_sHsOsOsS	47.90		0.95		0.0198	0.00	0.00	1
H_at_sC_at_sHsOsPsP	48.50		0.96		0.0197	0.00	0.00	1
H_at_sC_at_sHsOsPsS	47.20		0.94		0.0199	0.00	0.00	1
H_at_sC_at_sHsOsSsS	46.60		0.93		0.0200	0.00	0.00	1
H_at_sC_at_sHsPsPsP	47.60		0.95		0.0199	0.00	0.00	1
H_at_sC_at_sHsPsPsS	46.60		0.93		0.0200	0.00	0.00	1
H_at_sC_at_sHsPsSsS	45.60		0.92		0.0202	0.00	0.00	1
H_at_sC_at_sHsSsSsS	45.00		0.91		0.0202	0.00	0.00	1
H_at_sC_at_tCsH	43.36	0.40	0.83	0.01	0.0192	0.00	1.10	7
H_at_sC_at_tNsH	40.50		0.78		0.0193	0.00	0.00	1
H_at_sN_at_dCsH	33.07	3.36	0.63	0.06	0.0189	0.00	13.96	27
H_at_sN_at_dNsH	34.39	1.02	0.65	0.01	0.0189	0.00	3.73	7
H_at_sN_at_dOsH	39.90		0.70		0.0176	0.00	0.00	1
H_at_sN_at_dPsH	32.85	1.07	0.62	0.02	0.0189	0.00	4.24	6
H_at_sN_at_dSsH	33.80		0.64		0.0189	0.00	0.00	1
H_at_sN_at_dSsN	33.30		0.62		0.0187	0.00	0.00	1
H_at_sN_at_sCsCsH	31.28	1.84	0.60	0.04	0.0193	0.00	8.58	5
H_at_sN_at_sCsHsH	33.26	1.63	0.63	0.03	0.0190	0.00	6.34	256
H_at_sN_at_sCsHsN	29.62	3.16	0.57	0.06	0.0193	0.00	14.74	5
H_at_sN_at_sCsHsO	33.10		0.65		0.0195	0.00	0.00	1
H_at_sN_at_sCsHsP	33.00		0.64		0.0193	0.00	0.00	1
H_at_sN_at_sCsHsS	30.92	1.69	0.60	0.03	0.0193	0.00	7.51	6
H_at_sN_at_sHsHsH	34.63	0.06	0.65	0.00	0.0187	0.00	0.19	3
H_at_sN_at_sHsHsN	33.88	1.83	0.64	0.03	0.0189	0.00	6.79	68

H_at_sN_at_sHsHsO	33.16	0.43	0.64	0.01	0.0192	0.00	1.65	14
H_at_sN_at_sHsHsP	32.52	0.95	0.62	0.01	0.0190	0.00	3.66	66
H_at_sN_at_sHsHsS	31.68	0.70	0.61	0.01	0.0193	0.00	2.99	16
H_at_sN_at_sHsNsN	34.70		0.67		0.0193	0.00	0.00	1
H_at_sN_at_sHsNsO	32.20		0.64		0.0197	0.00	0.00	1
H_at_sN_at_sHsNsP	33.70		0.64		0.0191	0.00	0.00	1
H_at_sN_at_sHsNsS	32.40		0.63		0.0195	0.00	0.00	1
H_at_sN_at_sHsOsO	30.30		0.61		0.0200	0.00	0.00	1
H_at_sN_at_sHsOsP	31.80		0.62		0.0195	0.00	0.00	1
H_at_sN_at_sHsOsS	30.50		0.61		0.0198	0.00	0.00	1
H_at_sN_at_sHsPsP	31.70		0.62		0.0195	0.00	0.00	1
H_at_sN_at_sHsPsS	31.00		0.61		0.0195	0.00	0.00	1
H_at_sN_at_sHsSsS	30.00		0.59		0.0197	0.00	0.00	1
H_at_sO_at_sCsH	23.45	1.13	0.42	0.02	0.0181	0.00	6.17	129
H_at_sO_at_sHsH	24.40	0.00	0.44	0.00	0.0180	0.00	0.00	2
H_at_sO_at_sHsN	23.40	0.41	0.43	0.01	0.0183	0.00	2.32	33
H_at_sO_at_sHsO	23.20	0.62	0.42	0.01	0.0183	0.00	3.18	8
H_at_sO_at_sHsP	22.75	0.54	0.42	0.01	0.0182	0.00	3.43	37
H_at_sO_at_sHsS	22.94	0.78	0.42	0.01	0.0183	0.00	4.85	9
H_at_sP_at_dCsH	75.11	1.71	1.47	0.03	0.0196	0.00	3.13	27
H_at_sP_at_dNsH	75.95	1.05	1.48	0.01	0.0195	0.00	1.48	6
H_at_sP_at_dOsCsHsO	74.60		1.53		0.0205	0.00	0.00	1
H_at_sP_at_dOsH	79.60		1.48		0.0186	0.00	0.00	1
H_at_sP_at_dPsH	75.24	0.88	1.46	0.01	0.0194	0.00	1.28	7
H_at_sP_at_dSsH	76.70		1.47		0.0192	0.00	0.00	1
H_at_sP_at_sCsCsH	75.10		1.50		0.0200	0.00	0.00	1
H_at_sP_at_sCsHsH	74.10	1.07	1.47	0.01	0.0199	0.00	1.60	256
H_at_sP_at_sCsHsN	76.70		1.52		0.0198	0.00	0.00	1
H_at_sP_at_sCsHsO	74.20		1.50		0.0202	0.00	0.00	1
H_at_sP_at_sCsHsP	75.20		1.49		0.0198	0.00	0.00	1
H_at_sP_at_sCsHsS	73.40		1.49		0.0203	0.00	0.00	1
H_at_sP_at_sHsHsH	75.67	0.06	1.46	0.00	0.0193	0.00	0.08	3
H_at_sP_at_sHsHsN	74.70	1.21	1.48	0.01	0.0198	0.00	1.79	66
H_at_sP_at_sHsHsO	74.31	0.94	1.48	0.01	0.0199	0.00	1.32	14
H_at_sP_at_sHsHsP	74.87	0.51	1.47	0.01	0.0196	0.00	0.86	68
H_at_sP_at_sHsHsS	74.15	0.27	1.47	0.00	0.0198	0.00	0.36	14
H_at_sP_at_sHsNsN	73.00		1.52		0.0208	0.00	0.00	1
H_at_sP_at_sHsNsO	71.80		1.51		0.0210	0.00	0.00	1
H_at_sP_at_sHsNsP	76.00		1.50		0.0197	0.00	0.00	1
H_at_sP_at_sHsNsS	71.90		1.50		0.0209	0.00	0.00	1
H_at_sP_at_sHsOsO	70.80		1.50		0.0212	0.00	0.00	1
H_at_sP_at_sHsOsP	73.20		1.49		0.0204	0.00	0.00	1
H_at_sP_at_sHsOsS	70.70		1.49		0.0211	0.00	0.00	1
H_at_sP_at_sHsPsP	75.40		1.48		0.0196	0.00	0.00	1
H_at_sP_at_sHsPsS	72.90		1.48		0.0203	0.00	0.00	1
H_at_sP_at_sHsSsS	70.90		1.48		0.0209	0.00	0.00	1
H_at_sS_at_sCsH	52.42	1.58	1.01	0.02	0.0192	0.00	3.67	130

H_at_sS_at_sHsH	53.50	0.00	1.01	0.00	0.0189	0.00	0.00	2
H_at_sS_at_sHsN	52.87	1.49	1.00	0.01	0.0190	0.00	3.12	33
H_at_sS_at_sHsO	52.71	0.72	1.00	0.01	0.0189	0.00	1.58	7
H_at_sS_at_sHsP	52.69	0.53	1.00	0.01	0.0190	0.00	1.33	33
H_at_sS_at_sHsS	52.21	0.74	0.99	0.01	0.0190	0.00	1.64	8
N_at_dCsC	121.25	2.09	8.07	0.02	0.0665	0.00	1.75	44
N_at_dCsH	141.67	3.63	8.09	0.07	0.0571	0.00	2.72	27
N_at_dCsN	114.17	2.79	7.60	0.06	0.0666	0.00	2.55	6
N_at_dCsO	115.50	0.71	7.52	0.06	0.0651	0.00	1.04	2
N_at_dCsP	130.00		8.38		0.0645	0.00	0.00	1
N_at_dCsS	123.00	1.73	8.06	0.02	0.0655	0.00	1.42	3
N_at_dNsC	109.50	3.54	7.41	0.12	0.0676	0.00	3.61	2
N_at_dNsH	120.86	2.34	7.30	0.03	0.0604	0.00	1.99	7
N_at_dNsN	102.70	2.30	7.03	0.01	0.0685	0.00	2.25	4
N_at_dNsO	99.30		6.87		0.0692	0.00	0.00	1
N_at_dNsP	115.00		7.59		0.0660	0.00	0.00	1
N_at_dNsS	107.00		7.28		0.0680	0.00	0.00	1
N_at_dOsC	99.60		6.88		0.0691	0.00	0.00	1
N_at_dOsH	109.00		6.85		0.0628	0.00	0.00	1
N_at_dOsN	93.10		6.65		0.0714	0.00	0.00	1
N_at_dOsO	90.50		6.43		0.0710	0.00	0.00	1
N_at_dOsP	106.00		7.00		0.0660	0.00	0.00	1
N_at_dOsS	98.20		6.69		0.0681	0.00	0.00	1
N_at_dPsC	133.00		8.31		0.0625	0.00	0.00	1
N_at_dPsH	155.33	1.97	8.38	0.05	0.0539	0.00	1.42	6
N_at_dPsN	120.00		7.82		0.0652	0.00	0.00	1
N_at_dPsO	123.00		7.73		0.0628	0.00	0.00	1
N_at_dPsP	141.00		8.71		0.0618	0.00	0.00	1
N_at_dPsS	131.00		8.37		0.0639	0.00	0.00	1
N_at_dSsC	118.00		7.98		0.0676	0.00	0.00	1
N_at_dSsH	136.00		7.94		0.0584	0.00	0.00	1
N_at_dSsN	128.00	26.87	7.97	0.62	0.0622	0.01	22.37	2
N_at_dSsO	111.00		7.47		0.0673	0.00	0.00	1
N_at_dSsP	127.00		8.33		0.0656	0.00	0.00	1
N_at_dSsS	118.00		7.97		0.0675	0.00	0.00	1
N_at_sCsCsC	82.05	3.18	7.95	0.01	0.0969	0.00	3.88	2
N_at_sCsCsH	100.76	1.18	8.09	0.09	0.0803	0.00	1.60	5
N_at_sCsCsN	83.30		7.65		0.0918	0.00	0.00	1
N_at_sCsCsO	82.30		7.51		0.0913	0.00	0.00	1
N_at_sCsCsP	91.00		8.25		0.0907	0.00	0.00	1
N_at_sCsCsS	85.10		7.97		0.0937	0.00	0.00	1
N_at_sCsHsH	115.24	2.69	7.98	0.05	0.0693	0.00	2.41	128
N_at_sCsHsN	92.62	1.98	7.71	0.05	0.0833	0.00	2.24	5
N_at_sCsHsO	96.40		7.48		0.0776	0.00	0.00	1
N_at_sCsHsP	110.00		8.32		0.0756	0.00	0.00	1
N_at_sCsHsS	99.80	1.35	8.09	0.07	0.0810	0.00	1.64	6
N_at_sCsNsN	76.20		7.33		0.0962	0.00	0.00	1

N_at_sCsNsO	77.50		7.20		0.0929	0.00	0.00	1
N_at_sCsNsP	87.00		7.92		0.0910	0.00	0.00	1
N_at_sCsNsS	80.90		7.65		0.0946	0.00	0.00	1
N_at_sCsOsO	78.30		7.06		0.0902	0.00	0.00	1
N_at_sCsOsS	82.80		7.49		0.0905	0.00	0.00	1
N_at_sCsPsO	88.90		7.77		0.0874	0.00	0.00	1
N_at_sCsPsP	99.00		8.64		0.0873	0.00	0.00	1
N_at_sCsPsS	92.00		8.27		0.0899	0.00	0.00	1
N_at_sCsSsS	86.00		8.05		0.0936	0.00	0.00	1
N_at_sHsHsH	141.00		7.94		0.0563	0.00	0.00	1
N_at_sHsHsN	109.56	2.30	7.61	0.02	0.0695	0.00	2.11	34
N_at_sHsHsO	109.86	1.07	7.45	0.01	0.0678	0.00	0.99	7
N_at_sHsHsP	129.79	2.38	8.34	0.02	0.0643	0.00	1.86	33
N_at_sHsHsS	121.63	1.30	8.06	0.06	0.0662	0.00	1.28	8
N_at_sHsNsN	91.50		7.31		0.0799	0.00	0.00	1
N_at_sHsNsO	91.20		7.16		0.0785	0.00	0.00	1
N_at_sHsNsP	105.00		7.98		0.0760	0.00	0.00	1
N_at_sHsNsS	97.70		7.66		0.0784	0.00	0.00	1
N_at_sHsOsO	90.00		7.01		0.0779	0.00	0.00	1
N_at_sHsOsP	105.00		7.80		0.0743	0.00	0.00	1
N_at_sHsOsS	97.40		7.48		0.0768	0.00	0.00	1
N_at_sHsPsP	120.00		8.67		0.0723	0.00	0.00	1
N_at_sHsPsS	112.00		8.41		0.0751	0.00	0.00	1
N_at_sHsSsS	105.00		8.09		0.0770	0.00	0.00	1
N_at_sNsNsN	72.10		7.03		0.0975	0.00	0.00	1
N_at_sNsNsO	73.20		6.89		0.0941	0.00	0.00	1
N_at_sNsNsP	83.10		7.60		0.0915	0.00	0.00	1
N_at_sNsNsS	77.30		7.28		0.0942	0.00	0.00	1
N_at_sNsOsO	75.50		6.76		0.0895	0.00	0.00	1
N_at_sNsOsP	84.60		7.44		0.0879	0.00	0.00	1
N_at_sNsOsS	80.80		7.15		0.0885	0.00	0.00	1
N_at_sNsPsP	95.70		8.33		0.0870	0.00	0.00	1
N_at_sNsPsS	87.90		7.90		0.0899	0.00	0.00	1
N_at_sNsSsS	85.20		7.64		0.0897	0.00	0.00	1
N_at_sOsOsO	72.70		6.58		0.0905	0.00	0.00	1
N_at_sOsOsP	84.70		7.27		0.0858	0.00	0.00	1
N_at_sOsOsS	79.10		7.01		0.0886	0.00	0.00	1
N_at_sOsPsP	95.40		8.16		0.0855	0.00	0.00	1
N_at_sOsPsS	89.50		7.75		0.0866	0.00	0.00	1
N_at_sOsSsS	83.60		7.55		0.0903	0.00	0.00	1
N_at_sPsPsP	108.00		8.93		0.0827	0.00	0.00	1
N_at_sPsPsS	101.00		8.64		0.0855	0.00	0.00	1
N_at_sPsSsS	93.90		8.38		0.0892	0.00	0.00	1
N_at_sSsSsS	87.60		8.14		0.0929	0.00	0.00	1
N_at_tC	160.83	1.72	8.03	0.03	0.0499	0.00	1.12	6
O_at_dC	136.12	1.42	9.06	0.03	0.0665	0.00	1.11	43
O_at_dN	120.00	3.35	8.38	0.03	0.0698	0.00	2.82	6

O_at_dO	114.00	0.00	7.96	0.00	0.0698	0.00	0.00	2
O_at_dP	149.40	2.50	9.27	0.10	0.0621	0.00	1.98	10
O_at_dS	136.05	2.72	9.19	0.09	0.0676	0.00	2.21	20
O_at_sCsC	102.67	0.82	9.02	0.01	0.0879	0.00	0.81	15
O_at_sCsH	120.53	2.01	9.03	0.01	0.0750	0.00	1.67	129
O_at_sCsN	99.70	3.25	8.68	0.01	0.0871	0.00	3.26	2
O_at_sCsO	100.00		8.53		0.0853	0.00	0.00	1
O_at_sCsP	107.50	1.91	9.24	0.03	0.0859	0.00	1.81	4
O_at_sCsS	103.00	2.83	8.99	0.07	0.0873	0.00	2.86	2
O_at_sHsH	149.00		9.03		0.0606	0.00	0.00	1
O_at_sHsN	117.91	2.10	8.69	0.02	0.0737	0.00	1.80	33
O_at_sHsO	118.00	1.31	8.52	0.02	0.0722	0.00	1.14	8
O_at_sHsP	131.27	1.73	9.22	0.02	0.0703	0.00	1.33	37
O_at_sHsS	124.89	1.90	8.98	0.04	0.0719	0.00	1.59	9
O_at_sNsN	98.00		8.39		0.0856	0.00	0.00	1
O_at_sNsO	95.50		8.21		0.0860	0.00	0.00	1
O_at_sNsP	108.00		8.86		0.0820	0.00	0.00	1
O_at_sNsS	102.00		8.64		0.0847	0.00	0.00	1
O_at_sOsO	95.30		8.06		0.0846	0.00	0.00	1
O_at_sOsP	106.00		8.67		0.0818	0.00	0.00	1
O_at_sOsS	100.00		8.49		0.0849	0.00	0.00	1
O_at_sPsP	117.00		9.37		0.0801	0.00	0.00	1
O_at_sPsS	111.00		9.11		0.0821	0.00	0.00	1
O_at_sSsS	106.00		8.86		0.0836	0.00	0.00	1
P_at_dCsC	179.00		13.60		0.0760	0.00	0.00	1
P_at_dCsH	195.30	8.69	13.77	0.13	0.0705	0.00	4.55	27
P_at_dCsN	166.00		13.40		0.0807	0.00	0.00	1
P_at_dCsO	169.00		13.40		0.0793	0.00	0.00	1
P_at_dCsP	195.00		14.10		0.0723	0.00	0.00	1
P_at_dCsS	181.00		13.80		0.0762	0.00	0.00	1
P_at_dNsC	169.00		13.40		0.0793	0.00	0.00	1
P_at_dNsH	179.50	8.12	13.48	0.12	0.0751	0.00	4.60	6
P_at_dNsN	158.00		13.20		0.0835	0.00	0.00	1
P_at_dNsO	158.00		13.20		0.0835	0.00	0.00	1
P_at_dNsP	183.00		13.90		0.0760	0.00	0.00	1
P_at_dNsS	171.00		13.60		0.0795	0.00	0.00	1
P_at_dOsC	157.00		13.20		0.0841	0.00	0.00	1
P_at_dOsCsCsO	38.30		11.80		0.3081	0.00	0.00	1
P_at_dOsCsHsO	41.40		11.80		0.2850	0.00	0.00	1
P_at_dOsCsOsO	35.00		11.70		0.3343	0.00	0.00	1
P_at_dOsH	159.00		13.20		0.0830	0.00	0.00	1
P_at_dOsN	149.00		13.00		0.0872	0.00	0.00	1
P_at_dOsO	147.00		13.00		0.0884	0.00	0.00	1
P_at_dOsOsOsO	30.00		11.50		0.3833	0.00	0.00	1
P_at_dOsP	171.00		13.70		0.0801	0.00	0.00	1
P_at_dOsS	161.00		13.50		0.0839	0.00	0.00	1
P_at_dPsC	207.00		14.50		0.0700	0.00	0.00	1

P_at_dPsH	215.57	3.87	14.41	0.04	0.0669	0.00	1.81	7
P_at_dPsN	194.00		14.20		0.0732	0.00	0.00	1
P_at_dPsO	197.00		14.30		0.0726	0.00	0.00	1
P_at_dPsP	221.00		14.90		0.0674	0.00	0.00	1
P_at_dPsS	210.00		14.70		0.0700	0.00	0.00	1
P_at_dSsC	180.00		13.80		0.0767	0.00	0.00	1
P_at_dSsH	184.00		13.80		0.0750	0.00	0.00	1
P_at_dSsN	169.00		13.60		0.0805	0.00	0.00	1
P_at_dSsO	168.00		13.50		0.0804	0.00	0.00	1
P_at_dSsP	195.00		14.30		0.0733	0.00	0.00	1
P_at_dSsS	184.00		14.10		0.0766	0.00	0.00	1
P_at_sCsCsC	145.00		13.50		0.0931	0.00	0.00	1
P_at_sCsCsH	150.00		13.50		0.0900	0.00	0.00	1
P_at_sCsCsN	138.00		13.30		0.0964	0.00	0.00	1
P_at_sCsCsO	139.00		13.30		0.0957	0.00	0.00	1
P_at_sCsCsP	159.00		14.00		0.0881	0.00	0.00	1
P_at_sCsCsS	149.00		13.80		0.0926	0.00	0.00	1
P_at_sCsHsH	150.73	2.59	13.49	0.07	0.0895	0.00	1.80	128
P_at_sCsHsN	142.00		13.30		0.0937	0.00	0.00	1
P_at_sCsHsO	142.00		13.30		0.0937	0.00	0.00	1
P_at_sCsHsP	163.00		14.00		0.0859	0.00	0.00	1
P_at_sCsHsS	154.00		13.70		0.0890	0.00	0.00	1
P_at_sCsNsN	132.00		13.10		0.0992	0.00	0.00	1
P_at_sCsNsO	133.00		13.10		0.0985	0.00	0.00	1
P_at_sCsNsP	151.00		13.80		0.0914	0.00	0.00	1
P_at_sCsNsS	143.00		13.60		0.0951	0.00	0.00	1
P_at_sCsOsO	133.00		13.10		0.0985	0.00	0.00	1
P_at_sCsOsP	153.00		13.80		0.0902	0.00	0.00	1
P_at_sCsOsS	143.00		13.60		0.0951	0.00	0.00	1
P_at_sCsPsP	173.00		14.50		0.0838	0.00	0.00	1
P_at_sCsPsS	164.00		14.30		0.0872	0.00	0.00	1
P_at_sCsSsS	155.00		14.10		0.0910	0.00	0.00	1
P_at_sHsHsH	159.00		13.40		0.0843	0.00	0.00	1
P_at_sHsHsN	143.67	3.12	13.28	0.06	0.0924	0.00	2.22	33
P_at_sHsHsO	143.00	1.29	13.20	0.00	0.0923	0.00	0.90	7
P_at_sHsHsP	166.29	2.43	13.94	0.05	0.0838	0.00	1.50	34
P_at_sHsHsS	156.14	0.69	13.70	0.00	0.0877	0.00	0.44	7
P_at_sHsNsN	134.00		13.10		0.0978	0.00	0.00	1
P_at_sHsNsO	135.00		13.10		0.0970	0.00	0.00	1
P_at_sHsNsP	155.00		13.70		0.0884	0.00	0.00	1
P_at_sHsNsS	145.00		13.50		0.0931	0.00	0.00	1
P_at_sHsOsO	135.00		13.00		0.0963	0.00	0.00	1
P_at_sHsOsP	156.00		13.70		0.0878	0.00	0.00	1
P_at_sHsOsS	146.00		13.50		0.0925	0.00	0.00	1
P_at_sHsPsP	176.00		14.50		0.0824	0.00	0.00	1
P_at_sHsPsS	168.00		14.20		0.0845	0.00	0.00	1
P_at_sHsSsS	158.00		14.00		0.0886	0.00	0.00	1

P_at_sNsNsN	130.00		13.00		0.1000	0.00	0.00	1
P_at_sNsNsO	130.00		13.00		0.1000	0.00	0.00	1
P_at_sNsNsP	144.00		13.60		0.0944	0.00	0.00	1
P_at_sNsNsS	140.00		13.40		0.0957	0.00	0.00	1
P_at_sNsOsO	130.00		12.90		0.0992	0.00	0.00	1
P_at_sNsOsP	146.00		13.60		0.0932	0.00	0.00	1
P_at_sNsOsS	140.00		13.40		0.0957	0.00	0.00	1
P_at_sNsPsP	165.00		14.30		0.0867	0.00	0.00	1
P_at_sNsPsS	157.00		14.10		0.0898	0.00	0.00	1
P_at_sNsSsS	151.00		13.90		0.0921	0.00	0.00	1
P_at_sOsOsO	123.00		12.80		0.1041	0.00	0.00	1
P_at_sOsOsP	147.00		13.60		0.0925	0.00	0.00	1
P_at_sOsOsS	133.00		13.30		0.1000	0.00	0.00	1
P_at_sOsPsP	167.00		14.30		0.0856	0.00	0.00	1
P_at_sOsPsS	158.00		14.10		0.0892	0.00	0.00	1
P_at_sOsSsS	148.00		13.80		0.0932	0.00	0.00	1
P_at_sPsPsP	187.00		15.00		0.0802	0.00	0.00	1
P_at_sPsPsS	178.00		14.80		0.0831	0.00	0.00	1
P_at_sPsSsS	170.00		14.60		0.0859	0.00	0.00	1
P_at_sSsSsS	160.00		14.30		0.0894	0.00	0.00	1
S_at_dC	233.57	11.14	15.65	0.16	0.0670	0.00	4.87	28
S_at_dN	223.33	6.53	15.33	0.10	0.0687	0.00	3.00	6
S_at_dNdOsCsN	113.00		14.40		0.1274	0.00	0.00	1
S_at_dO	208.00		15.00		0.0721	0.00	0.00	1
S_at_dOdOsCsC	56.05	0.35	13.65	0.07	0.2435	0.00	0.82	2
S_at_dOdOsCsN	50.33	0.31	13.23	0.06	0.2629	0.00	0.75	3
S_at_dOdOsCsO	49.13	0.72	13.17	0.06	0.2680	0.00	1.54	3
S_at_dOsCsN	116.00		14.40		0.1241	0.00	0.00	1
S_at_dOsCsNsN	54.50		13.50		0.2477	0.00	0.00	1
S_at_dP	257.00	3.16	16.57	0.05	0.0645	0.00	1.27	6
S_at_dS	233.00	0.00	15.90	0.00	0.0682	0.00	0.00	2
S_at_sCsC	195.50	9.19	15.80	0.14	0.0808	0.00	4.79	2
S_at_sCsH	205.61	3.71	15.85	0.07	0.0771	0.00	1.86	130
S_at_sCsN	182.33	10.12	15.40	0.17	0.0845	0.00	5.66	3
S_at_sCsO	192.00		15.50		0.0807	0.00	0.00	1
S_at_sCsP	211.00		16.20		0.0768	0.00	0.00	1
S_at_sCsS	200.00		15.90		0.0795	0.00	0.00	1
S_at_sHsH	225.00		15.80		0.0702	0.00	0.00	1
S_at_sHsN	199.06	3.98	15.57	0.07	0.0782	0.00	2.05	33
S_at_sHsO	197.43	2.15	15.43	0.05	0.0781	0.00	1.13	7
S_at_sHsP	220.21	2.52	16.12	0.04	0.0732	0.00	1.18	33
S_at_sHsS	211.38	2.39	15.91	0.04	0.0753	0.00	1.15	8
S_at_sNsN	183.00		15.20		0.0831	0.00	0.00	1
S_at_sNsO	178.00		15.10		0.0848	0.00	0.00	1
S_at_sNsP	202.00		15.90		0.0787	0.00	0.00	1
S_at_sNsS	189.00		15.50		0.0820	0.00	0.00	1
S_at_sOsO	176.00		15.00		0.0852	0.00	0.00	1

S_at_sOsP	198.00	15.70	0.0793	0.00	0.00	1
S_at_sOsS	187.00	15.50	0.0829	0.00	0.00	1
S_at_sPsP	220.00	16.40	0.0745	0.00	0.00	1
S_at_sPsS	209.00	16.20	0.0775	0.00	0.00	1
S_at_sSsS	199.00	15.90	0.0799	0.00	0.00	1
